# Enhancement of the Precision ID Mitochondrial DNA Whole Genome System for Challenging Unidentified Human Remains

**DOI:** 10.3390/genes16020119

**Published:** 2025-01-22

**Authors:** Lauren C. Canale, Mavis Date-Chong, Jeanette Wallin, Sandra Sheehan, Jessica Battaglia, Michelle Halsing, Daniela Cuenca

**Affiliations:** California Department of Justice, Jan Bashinski DNA Laboratory, Richmond, CA 94804, USA; mavisd.chong@doj.ca.gov (M.D.-C.); jeanette.wallin@doj.ca.gov (J.W.); sandra.sheehan@doj.ca.gov (S.S.); jessica.battaglia@doj.ca.gov (J.B.); michelle.halsing@doj.ca.gov (M.H.); daniela.cuenca@doj.ca.gov (D.C.)

**Keywords:** massively parallel sequencing, mitochondrial DNA, inhibition, forensics, BSA, microbial DNA, next-generation sequencing

## Abstract

Background: The Precision ID mitochondrial (mt) DNA Whole Genome system is a fully automated massively parallel sequencing (MPS) solution for the whole mitochondrial genome. While extremely sensitive, the Precision ID system is susceptible to inhibitors and microbial DNA that are often co-extracted from human remains. Methods: DNA templates spiked with varying amounts of hematin, humic acid, and calcium, along with bones containing degraded and non-human DNA, were sequenced using the Precision ID system with and without the addition of bovine serum albumin (BSA). Results: BSA added to the initial PCR reaction successfully improved the robustness of the Precision ID system while not negatively impacting the sequencing success of uninhibited samples. The success of BSA is inhibitor-concentration dependent and is effective for templates containing at least 50 ng/μL humic acid, 50 μM hematin, and 1500 μM calcium ions. Furthermore, the presence of microbial DNA in addition to an inhibitor, results in non-specific adaptor ligation to the non-human DNA; BSA can alleviate the inhibition, allowing the human mtDNA to be amplified and sequenced. Conclusions: The addition of BSA to the Precision ID mtDNA system can yield successful sequencing results from challenging case samples that would otherwise fail.

## 1. Introduction

Sequence analysis of mitochondrial DNA (mtDNA) is routinely used in forensic laboratories for challenging or compromised sample types, such as bones, teeth, and hair shafts, whose nuclear DNA (nDNA) content is too degraded for conventional capillary electrophoresis short tandem repeat (CE-STR) analysis [1,2,3,4,5]. Sanger sequencing has traditionally been used to sequence the control region of the mitochondrial genome (mitogenome); however, over the past decade, laboratories have transitioned to sequencing the whole mitogenome due to the increased discrimination potential [6,7]. Massively parallel sequencing (MPS) technologies allow for relatively quick routine analysis of the whole mitogenome and also nuclear SNP and forensic STR analysis from challenging sample types due to increased sensitivity, multiplexing capabilities, and the ability to be fully automated [8,9,10]. These advancements have led to a gradual implementation of MPS in public forensic laboratories over the past decade, including the California Department of Justice (CA DOJ), Bureau of Forensic Services, Missing Persons DNA Program (MPDP) [11].

The Applied Biosystems Precision ID system (ThermoFisher; Waltham, MA, USA) is a fully automated MPS solution, consisting of the Precision ID mtDNA Whole Genome Panel, Ion Chef^TM^ instrument, Ion S5^TM^ semiconductor sequencing system, and Torrent Suite Software 5.10.0. The panel targets the whole mitogenome using 162 amplicons of an average length of 163 base pairs (bps). This system has been successfully validated and implemented for the analysis of challenging, degraded forensic samples as well as routine samples that benefit from lineage marker analysis [11,12,13,14,15], and has been utilized by the MPDP since 2020. Despite the greater sensitivity and smaller target size, the Ion system is susceptible to inhibitors, often co-extracted with human remains, including calcium, collagen, hematin, humic acid, and melanin [11,16]. The Precision ID mtDNA MPS workflow requires several polymerase chain reaction (PCR) steps from library preparation to sequencing; the initial PCR, during which the targets of interest are amplified, is extremely critical in determining the sequencing success downstream.

Polymerases used in a variety of MPS systems have been shown to be sensitive to inhibition [11,16,17,18]. Bovine serum albumin (BSA) is a known PCR enhancer capable of improving DNA polymerization in the presence of a range of inhibitory molecules [18,19,20]. Focusing on bone-derived PCR inhibitors (collagen, calcium, and humic acid), Eilert et al. [19] demonstrated that different polymerases vary in their susceptibility to inhibition and that BSA successfully reversed the inhibitory effect on most of the polymerases. Using the ForenSeq^TM^ DNA Signature Prep MPS system (Verogen; San Diego, CA, USA), Sidstedt et al. [18] added 10 μg of BSA per amplification reaction and determined that BSA increased the ForenSeq^TM^ system’s tolerance of humic acid and hematin-inhibited samples. This is a simple solution to remediate inhibited samples examined with MPS technology and may be beneficial to other systems in addition to the ForenSeq^TM^ system.

**Figure 1:**
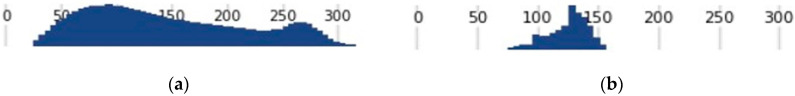
Precision ID mtDNA Whole Genome Panel read-length histograms. (**a**) A failed inhibited bone sample containing inhibitor and non-human co-extracted DNA. (**b**) A successfully sequenced sample.

The main objective of this study was to determine the impact of BSA on the Precision ID mtDNA Whole Genome system when added during automated library preparation of challenging missing and unidentified persons case samples. This was tested using templates spiked with hematin, humic acid, and calcium, and non-probative casework bones previously established to contain degraded DNA and non-human DNA. Using the Precision ID system with inhibited bone samples, MPDP analysts have observed read-length histograms generated by the Torrent Suite Software 5.10.0 showing amplicon sizes ranging from <50 to ~300 base pairs (bps) that appear as a “whale-pattern”, illustrated in Figure 1a.

This is inconsistent with the expected Precision ID mtDNA amplicon range of ~75–150 bps (Figure 1b) obtained for successfully sequenced samples. Although a high number of sequencing reads were generated for those bone samples (up to >1 million), often <1% of the reads were used and aligned with the human mitogenome, and no meaningful sequence data was obtained. Cuenca et al. [11] experienced similar results with two bone samples during the validation and speculated that the abnormal size range of the amplicons and the high number of unaligned reads were suggestive of non-specific amplification products, possibly of microbial origin. Another goal of this study was to further investigate this theory.

## 2. Materials and Methods

### 2.1. Sample Information

#### 2.1.1. Control Buccal, Bone, and Microbial DNA Samples

Two buccal samples collected from laboratory personnel with informed consent were used for the inhibition (QC) and sensitivity studies (PS1). These samples were extracted with the AutoMate Express^TM^ Forensic DNA Extraction System (ThermoFisher; Waltham, MA, USA) and the PrepFiler^®^ Express BTA^TM^ Forensic DNA Extraction Kit (ThermoFisher; Waltham, MA, USA) according to the manufacturer’s protocols [21,22].

Bone1 is a quality control femur bone sample obtained from the University of California, Davis, Body Donation Program. A portion of Bone1, used during the sensitivity experiments for this study, was extracted with the demineralization method described by Amory et al. [23] with one deviation; an Amicon Ultra-15 30K (MilliporeSigma; Burlington, MA, USA) was used instead of a 100K filtration device.

Autosomal DNA from the resulting extracts of QC, PS1, and Bone1 was quantified using the QuantiFiler^TM^ Trio DNA Quantitation Kit (ThermoFisher; Waltham, MA, USA on the QuantStudio^TM^ 5 Real-Time PCR system (ThermoFisher; Waltham, MA, USA) following the manufacturer’s protocol [24]. *Staphylococcus epidermidis* microbial DNA, purchased from the American Type Culture Collection (Manassas, VA, USA), was quantified using the QuantiFluor^®^ ONE dsDNA System (Promega; Madison, WI, USA) and the Quantus^TM^ Fluorometer (Promega; Madison, WI, USA), as described in the manufacturer’s technical manuals [25,26].

#### 2.1.2. Bone Casework Samples

Eighteen authentic non-probative bone casework samples from the CA DOJ MPDP exhibiting severe degradation/damage, inhibition, low template human DNA, and/or potential presence of non-human DNA were selected for Precision ID mtDNA re-analysis with BSA. Samples that did not have sufficient extract volume available were re-extracted following the same procedure as Bone1 described above. Bone powder amounts and extract volumes used were similar to their previous casework analyses; the template volumes and other sample description information are noted in Table S1, Supplementary Materials. Autosomal DNA was quantified using the QuantiFiler^TM^ Trio Kit [24] on the QuantStudio^TM^ 5 with no signs of inhibition per the delta IPC and amplification curves; however, only 2 μL of the sample was quantified. The template DNA quantity was determined by taking the geometric mean of the large and small Quantifiler^TM^ targets unless otherwise denoted. The total DNA amount (including potential microbial DNA) was measured using the QuantiFluor^®^ ONE dsDNA System and the Quantus^TM^ Fluorometer [25,26].

### 2.2. MPS Precision ID Workflow

MPS library preparation, quantitation, clonal amplification (templating), and sequencing were performed as described by Cuenca et al. [11]. Automated library preparation with the Precision ID DL8 Kit (ThermoFisher; Waltham, MA, USA) and the Precision ID mtDNA Whole Genome Panel (ThermoFisher; Waltham, MA, USA) was performed using the Ion Chef^TM^ instrument (ThermoFisher; Waltham, MA, USA). Pooled libraries were quantified with the Ion Library TaqMan^TM^ Quantitation Kit (ThermoFisher; Waltham, MA, USA) on the 7500 Real-Time PCR system (ThermoFisher; Waltham, MA, USA) per the manufacturer’s protocol [27] and diluted to 50 pM for template preparation [11]. Fully automated template preparation, enrichment, and chip-loading on the 530^TM^ Chip (ThermoFisher; Waltham, MA, USA) was performed with the Ion S5^TM^ Precision ID Chef and Sequencing Kit (ThermoFisher; Waltham, MA, USA) on the Ion Chef^TM^ instrument. The prepared chips were run on the Ion S5^TM^ system (ThermoFisher; Waltham, MA, USA) with a sequencing read length of 200 bps and 500 cycles. Bioinformatic data analysis was performed using the Torrent Suite Software 5.10.0 and HIDGenotyper 2.2 plug-in (ThermoFisher; Waltham, MA, USA). Analysis settings were varied based on how many libraries were pooled together; those settings can be found in Table 1. Sequence data was visually inspected with the Mito Integrative Genomics Viewer (IGV) version 1.09b (IGV Team; San Diego, CA, USA and Cambridge, MA, USA) [28]. The entire automated process is collectively referred to as the PID mtDNA system throughout this paper.

### 2.3. The Addition of BSA

BSA Fraction V (Calbiochem; San Diego, CA, USA) was prepared to a stock concentration of 50 mg/mL. Then, 10, 20, and 40 μg of BSA per amplification reaction were evaluated in the initial inhibition experiments; based on those results, 20 μg was selected for the remainder of the study. With the PID mtDNA system, there are three components to which BSA could be added: the template (column one of the amplification plate), the amplification reaction wells (columns two and three), or the primer pools (position A and B of the DL8 reagent cartridge). For 20 μg of BSA per amplification, the following volumes and concentrations were used: when adding BSA to the template, 1 μL of the 50 mg/mL stock was added to bring the total template volume to 15 μL; when adding BSA to the reaction wells, the stock was diluted to 20 mg/mL and 1 μL of the dilution was pipetted into each well in columns two and three of the amplification plate; when adding BSA directly to the primer pools, 6 μL of the 50 mg/mL stock was added to each primer pool tube for a total volume of 150 μL per tube. The template, amplification wells, and primer pools will each be referred to as the source of BSA when discussing the addition of BSA to the PID mtDNA system.

### 2.4. Sensitivity and Efficiency Studies

Sensitivity studies were performed to evaluate whether the addition of BSA would affect the efficiency and sensitivity of the PID mtDNA system. Three input amounts (100, 10, and 1 pg) of PS1 and Bone1 were targeted. Library preparation was performed in duplicate with BSA (20 μg) added to the primer pools, and in singlet with the standard manufacturer protocol (no BSA). The three libraries were pooled (24 multiplexed samples) for templating and sequencing. The MPDP has two Ion Chef^TM^ instruments (1CHEF and 2CHEF) and two Ion S5^TM^ systems (1S5 and 2S5); therefore, the sensitivity and efficiency of both instrument sets were assessed. The library preparation, templating, and sequencing process was conducted twice overall, once on each set of instruments (1CHEF/1S5 and 2CHEF/2S5), for a total of six prepared libraries and two sequenced pools (three libraries pooled per instrument set). The two instrument sets are referred to as 1S5 and 2S5 for this manuscript.

### 2.5. Inhibitors

Humic acid, hematin, and calcium (Ca^2+^) were prepared and tested at varying concentrations for respective inhibitory effects. Humic acid (Alfa Aesar; Haverhill, MA, USA) and calcium chloride (Sigma Aldrich; St. Louis, MO, USA) stock solutions were prepared in deionized water to a concentration of 700 ng/μL and 1 M, respectively. Hematin (Sigma Aldrich; St. Louis, MO, USA) was dissolved in 1 N NaOH and then diluted to 2000 μM with sterile water. The inhibitors were added to the template DNA for a total volume of 15 μL. During the amplification reaction set-up, the Ion Chef^TM^ transfers 6 μL of the template from column one of the IonCode plate to columns two and three for the amplification reactions; once the primer pools are transferred to those columns, the final library amplification volume is 20 μL. The final concentrations of the inhibitors in the individual amplification reactions were as follows: 1, 7.5, 15, 25, and 50 ng/μL humic acid; 3, 10, 20, and 50 μM hematin; 500 and 1500 μM Ca^2+^.

## 3. Results and Discussion

### 3.1. Sensitivity and Inhibition

**Figure 2:**
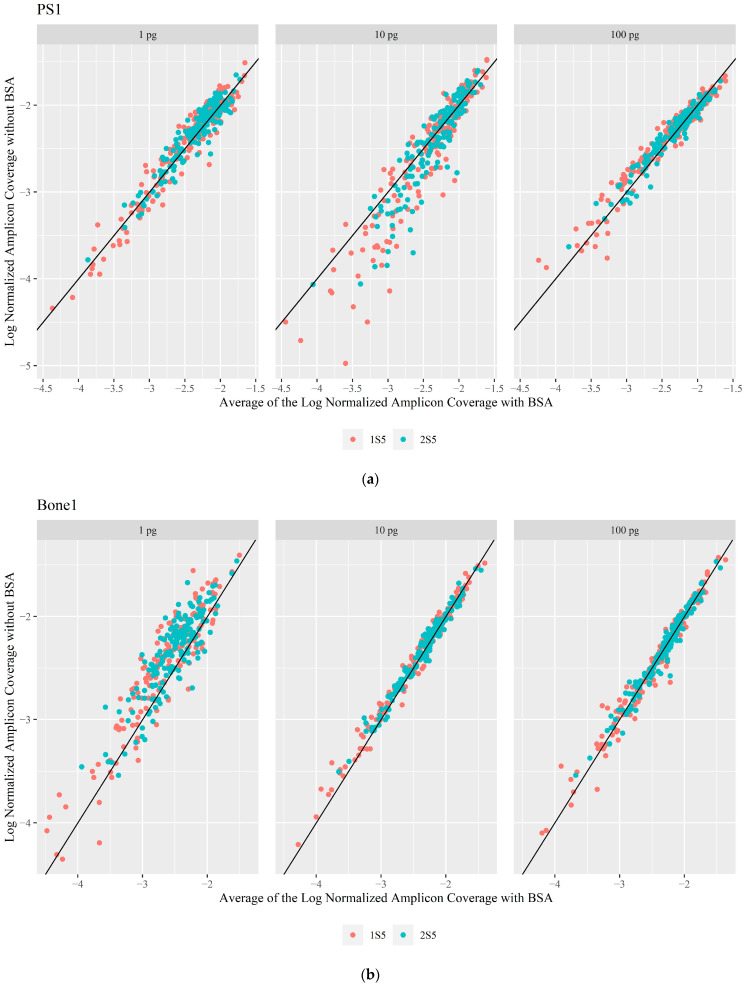
Pairwise plots of the log normalized amplicon coverage without BSA compared to with BSA for (**a**) PS1 and (**b**) Bone1. Due to unequal sample numbers (n_BSA_ = 12, n_No BSA_ = 6 for both PS1 and Bone1), the average of the log normalized coverage was taken for the BSA duplicates. The solid black line is the line of equivalence. Data points below the line are amplicons that performed better with the addition of BSA and points above the line are amplicons that performed better without the addition of BSA.

The effectiveness of BSA in relieving inhibition of the PID mtDNA system from varying amounts of humic acid, hematin, and calcium was assessed by evaluating haplotype concordance for QC and sequence data quality—coverage of reads across the mitogenome and percentage of usable reads mapped to the mitogenome—of each sample without and with the addition of BSA. The PID mtDNA system was not adversely affected by BSA without an inhibitor (Table 2; Table S2, Supplementary Materials), as the QC haplotype was fully concordant, and more than 98.42% of the mitogenome was above the coverage threshold (CT) regardless of the amount of BSA added during library preparation. Furthermore, the sensitivity and efficiency of the PID mtDNA system with and without BSA were evaluated by sequencing another buccal sample (PS1) and a bone sample (Bone1) at varying input template amounts (100, 10, and 1 pg). The analyzed haplotypes for PS1 and Bone1 were fully concordant with differences in the amplicon coverage. The Bone1 sequences at all input amounts, regardless of the addition of BSA, also contained nuclear mtDNA segments (NUMTs), which were filtered out during the analysis process. A pairwise analysis of the log normalized read depth (the log of the median number of reads per amplicon/total number of reads) of each of the 162 amplicons, obtained on both 1S5 and 2S5, showed tight clustering of the data points with and without BSA for both instrument sets (Figure 2). PS1 experienced a greater difference between BSA and no BSA at 10 pg due to an outlier sample that performed worse without BSA (Figure 2a; Figure S1a, Supplementary Materials). Bone1 showed a greater spread at 1 pg (Figure 2b) compared to 1 pg of PS1 (Figure 2a); the degradation index for Bone1 is greater than that for PS1 (5.63 and 0.75, respectively), which could contribute to this apparent lower sensitivity for Bone1. Template amounts were based on nuclear DNA from the different tissue types. As has been reported, nuclear DNA yields and mitochondrial DNA copy numbers vary between individuals and tissues, and between different parts of the same skeletal element [29,30,31,32,33]. Overall, BSA was shown to have a minimal negative impact on the amplification and library preparation efficiency of the PID mtDNA system. Differences in amplicon coverage, a slightly lower percentage of the mitogenome reported, and a lower percentage of mapped and used reads were observed when BSA was added—without additional inhibitors—as the sensitivity limit was approached; however, it was not considered consequential enough to be a concern. Additional data on the sensitivity and efficiency of the PID mtDNA system can be seen in the Supplementary Materials (Figures S1–S4 and Tables S3 and S4, Supplementary Materials).

**Figure 3:**
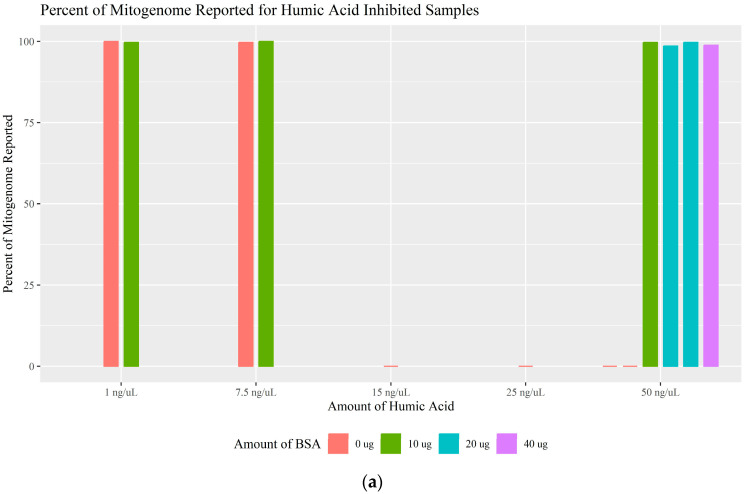

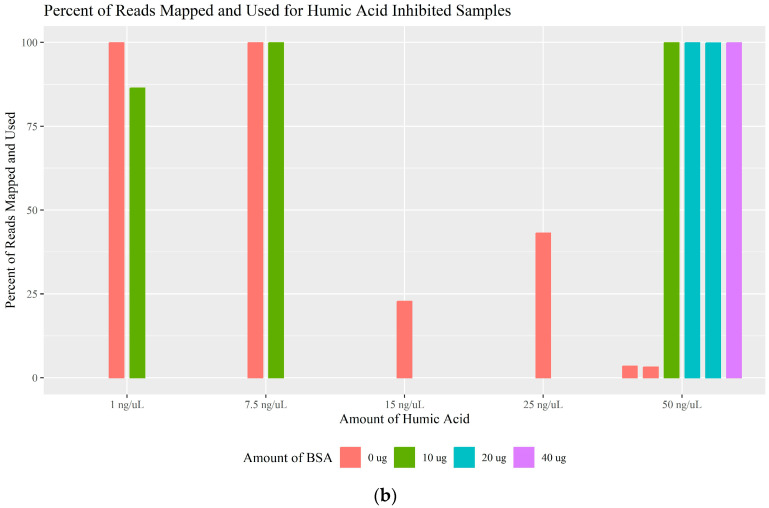
(**a**) The percentage of the mitogenome reported for each amount of humic acid tested separated by the BSA amount used. Complete inhibition/0% of the mitogenome reported is represented by a line at 0%. (**b**) The percentage of reads mapped and used for each amount of humic acid separated by the BSA amount used.

Humic acid is an organic compound found in soil and sediment that can interact with and bind the template DNA and the polymerase [34,35,36]. Based on previous studies, it is suggested that the primary mechanism of PCR inhibition is a direct effect on the DNA polymerase, decreasing the amplification efficiency, and eventually leading to complete inhibition [18,36,37]. Humic acid was tested at concentrations of 1, 7.5, 15, 25, and 50 ng/μL; sequencing results can be found in Table S5, Supplementary Materials. Overall, the higher concentrations of 15, 25, and 50 ng/μL caused full inhibition and the addition of BSA effectively overcame that inhibition. For the 50 ng/μL samples, more than 98.58% of the mitogenome was reported (Figure 3a) and at least 99.81% of the generated reads were mapped to the mitogenome and used (Figure 3b). Without the addition of BSA, 1 ng/μL of humic acid was not inhibitory while 7.5 ng/μL initially appeared to be partially inhibitory due to the read depth of the amplicon encompassing SNPs 263G and 295T (amplicon mt_3) dropping below the CT. However, during validation, amplicon mt_3 was identified to have low efficiency due to variant 228A causing a primer binding issue. Amplicon mt_3 also dropped out when QC was sequenced alone without both BSA and inhibitors. Therefore, it is likely that samples experiencing drop-out of only amplicon mt_3, such as the 7.5 ng/μL sample, are not inhibited but are instead experiencing the primer binding issue. When fully inhibited, the total number of reads generated for QC was less than 2900, with at most 1228 of 2847 being mapped and used (Figure S5; Table S5, Supplementary Materials). Supplementary Figure S5 also shows that, when inhibition is remediated with BSA, there is an almost linear relationship between the total number of reads and the number of reads mapped and used. The read-length histograms generated by the Torrent Suite Software 5.10.0 visually show the poor sequencing quality caused by inhibitory humic acid concentrations when compared to the samples for which BSA successfully reversed the effects of inhibition (Table 2; Table S5, Supplementary Materials). Amounts of BSA greater than 10 μg did not further enhance the performance of the system when 50 ng/μL of humic acid was present; however, BSA was capable of relieving almost all inhibition caused by humic acid.

**Figure 4:**
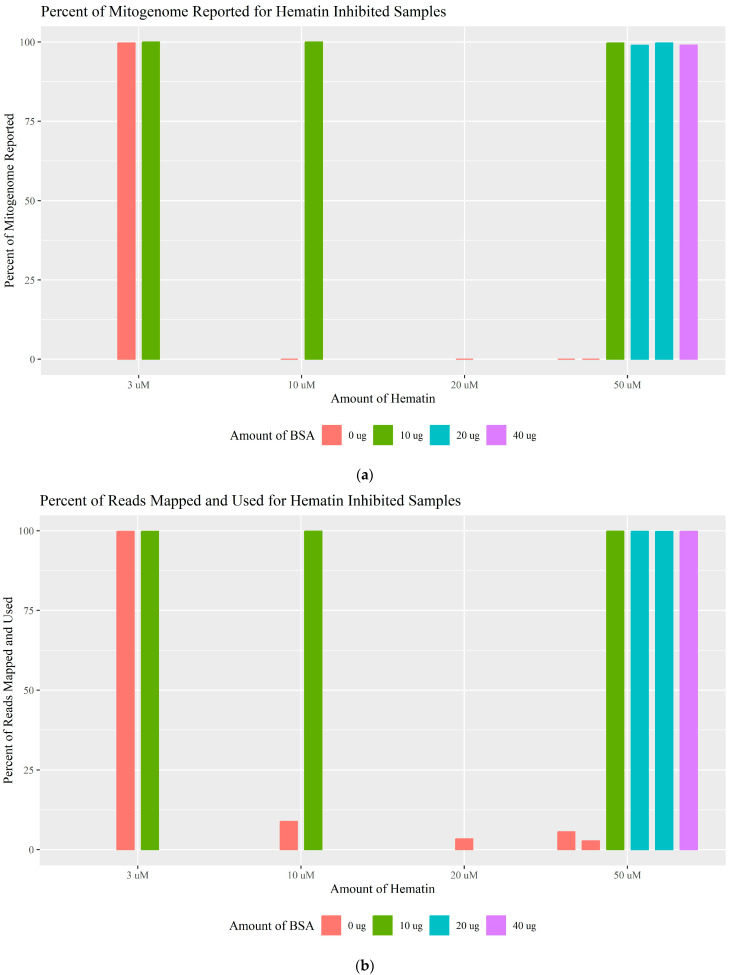
(**a**) The percentage of the mitogenome reported for each amount of hematin tested separated by the BSA amount used. Complete inhibition/0% of the mitogenome reported is represented by a line at 0%. (**b**) The percentage of reads mapped and used for each amount of hematin separated by the BSA amount used.

Hematin is a derivative of the heme group and is a useful model for blood in PCR inhibition experiments. Previous studies have shown that hematin causes lowered DNA polymerase activity, impacting the amplification efficiency, similar to humic acid [34,36,38]. Hematin was tested at concentrations of 3, 10, 20, and 50 μM (Table S5, Supplementary Materials). Without the addition of BSA, slight inhibition was observed at 3 μM with 93.10% of the mtDNA profile and 99.70% of the whole mitogenome reported, while 10, 20, and 50 μM were completely inhibitory, resulting in 0% of the mitogenome above the CT and less than 9% of the generated reads mapped and used (Figure 4). BSA improved the PCR efficiency for 3, 10, and 50 μM (20 μM was not tested with BSA), resulting in 100% haplotype concordance and 100% of the whole mitogenome reported for 3 and 10 μM. At 50 μM, 93.10% of the expected polymorphisms were recovered, more than 98.96% of the whole mitogenome was above the CT, and more than 99.77% of the reads were mapped and used regardless of the amount of BSA added. The same mt_3 amplicon fell below the threshold for all the hematin samples that did not generate complete profiles. This could be due to inhibition, or the primer binding issue described previously. Similar to humic acid, a very low number of reads was generated when only hematin was present, and overall, the total number of reads compared to the number of reads mapped and used revealed an almost linear relationship when inhibition was not present (Figure S6, Supplementary Materials). The read-length histograms for the inhibited hematin samples were similar to those of the humic acid-inhibited samples (Table 2; Table S5, Supplementary Materials); this is expected due to their similar PCR inhibition mechanisms.

**Figure 5:**
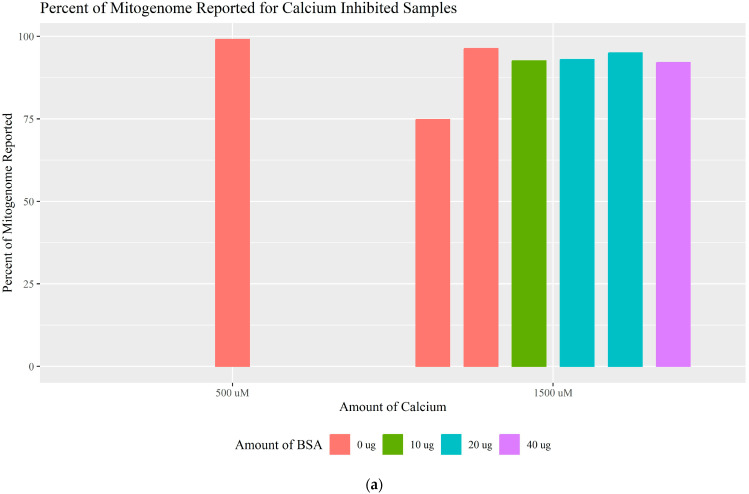

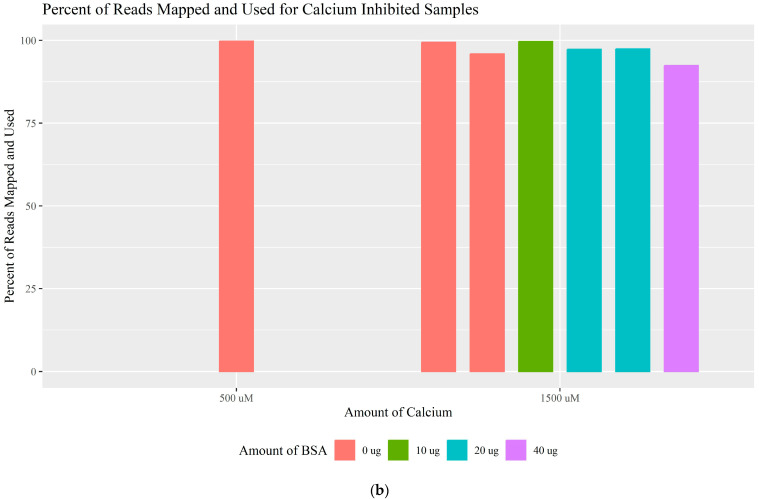
(**a**) The percentage of the mitogenome reported for each amount of calcium tested separated by the BSA amount used. (**b**) The percentage of reads mapped and used for each amount of calcium separated by the BSA amount used.

Calcium ions (Ca^2+^) are an inorganic substance found in bones that can inhibit polymerase activity by competing with magnesium, reducing the reaction efficiency [34,35,36]. Ca^2+^ concentrations of 500 and 1500 μM were tested with and without BSA. Ca^2+^ at 500 μM was not inhibitory (100% haplotype concordance and 99.08% of the whole mitogenome) and 1500 μM caused partial inhibition (72.41–96.55% complete haplotype and 74.81–96.29% of the whole mitogenome) (Figure 5a; Table S5, Supplementary Materials). The PID mtDNA system appeared to be more tolerant of Ca^2+^ as neither concentration was completely inhibitory; however, the sequencing results do suggest that the amplification efficiency of the panel was affected. Ca^2+^ has a different mechanism of inhibition than humic acid and hematin, which could explain the differences in both the amplicon coverage when partially inhibited (Figure S8, Supplementary Materials) and the read-length histograms (Table 2; Table S5, Supplementary Materials). The addition of BSA to calcium-affected samples did not result in a complete recovery of the expected polymorphisms or whole mitogenome coverage. At most, 96.55% of the haplotype and 94.95% of the mitogenome reached a read depth greater than the CT when 20 μg of BSA was added to the primer pools. Figure 5b and Supplementary Figure S7 further illustrate the tolerance to Ca^2+^ as indicated by the high numbers of reads generated (>500,000) and used (>92.40%), regardless of the addition of BSA. These results support the notion that BSA is capable of relieving the majority of inhibition caused by these common inhibitors but that increasing the amount of BSA does not increasingly enhance the PCR amplification.

### 3.2. Non-Human DNA

**Figure 6:**
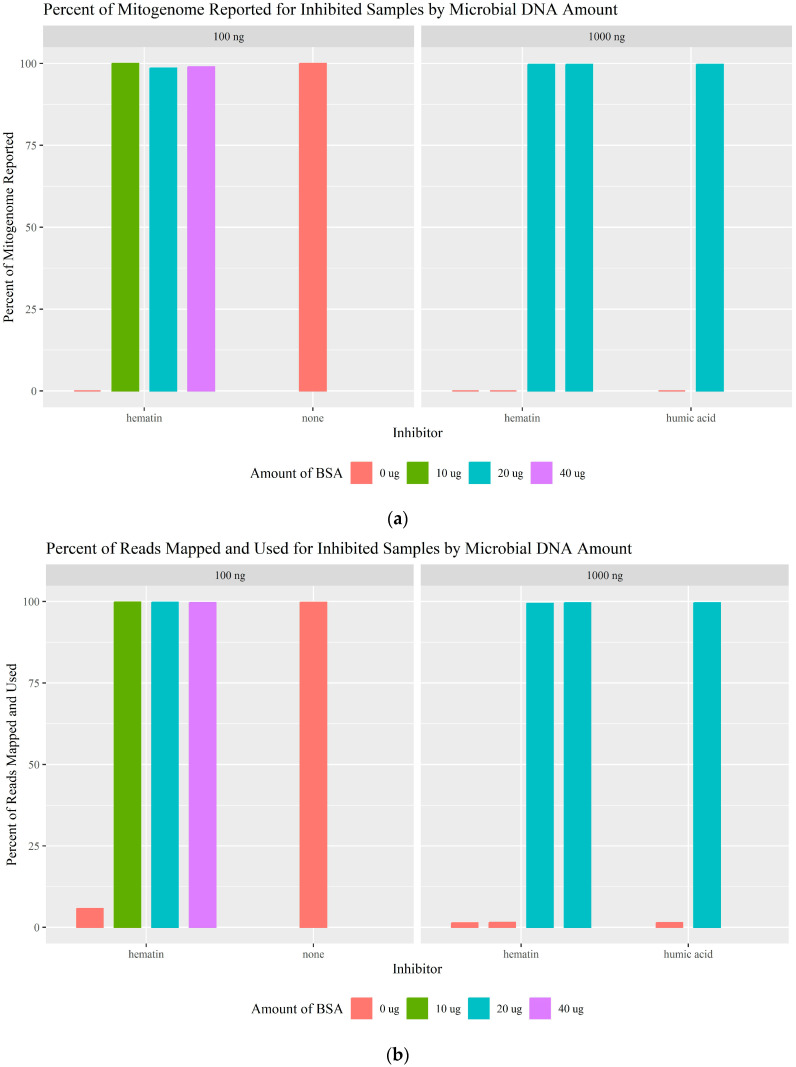

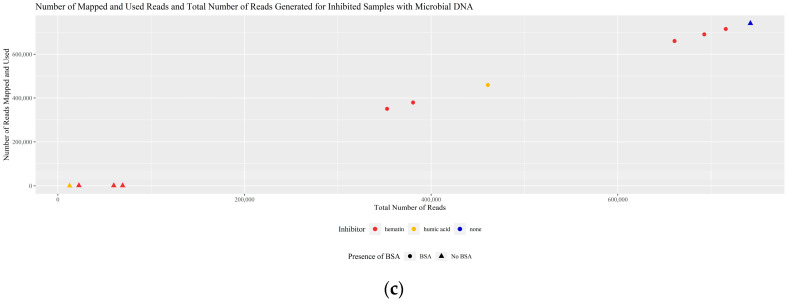
(**a**) The percentage of the mitogenome reported for each microbial DNA amount and added inhibitor. Complete inhibition/0% of the mitogenome reported is represented by a line at 0%. (**b**) The percentage of reads mapped and used for each microbial DNA amount and added inhibitor. (**c**) Scatter plot of the number of reads mapped and used compared to the total number of reads generated for each added inhibitor.

The inhibitory effect observed by the MPDP for samples with high numbers of reads, but the majority not aligning to the mitogenome was suspected to be caused by the presence of non-human, possibly microbial DNA [11]. In an attempt to replicate the unique “whale-pattern” read-length histogram shape seen with these types of samples, *S. epidermidis* microbial DNA was added to the QC extract with and without additional inhibitors. mtDNA sequencing of QC (100 pg) was not affected by the addition of 100 ng of microbial DNA alone (1000-fold more microbial DNA); 100% of the haplotype was concordant, and 100% of the whole mitogenome was reported (Figure 6a; Table S6, Supplementary Materials). However, when an inhibitor (humic acid [50 ng/μL] or hematin [10 and 50 μM]) was added to the QC extract with either 100 or 1000 ng of microbial DNA, complete PCR inhibition occurred (Figure 6a), and the histograms displayed the “whale-pattern” previously observed by MPDP (Table 3). While complete inhibition is expected for these amounts of humic acid and hematin (Section 3.1), a unique difference due to the addition of microbial DNA was observed; the total number of reads generated, while lower than the uninhibited samples, was higher than the inhibitor-only samples (Section 3.1; Figures S5 and S6, Supplementary Materials). However, the percentage of these reads mapped and used was less than 5.78% (Figure 6b; Table S6, Supplementary Materials). Figure 6c illustrates this phenomenon with large numbers of reads generated, but only a minimal number of the reads were aligned and used. Contrary to what was previously hypothesized [11], this indicates that this observation is not due to non-specific amplification but rather non-specific adaptor ligation. If amplification of the target DNA fails, then the adaptors will ligate to any available DNA in the sample, including nhDNA, and be subsequently sequenced. When the majority of the sample is nhDNA, a large number of reads will still be generated, but the number of human mtDNA reads will be significantly less. By testing different levels of microbial DNA, inhibitors, and the combination of both, we found that microbial DNA alone does not cause inhibition or non-specific amplification, but it is the combination of an inhibitor and microbial DNA in the sample that results in this read pattern.

With the addition of BSA, inhibition caused by the inhibitor in the presence of nhDNA was successfully overcome, with more than 99.45% of the reads mapped and used (Figure 6; Table 3; Table S6, Supplementary Materials). BSA was successful regardless of the amount of microbial DNA (100 or 1000 ng), the amount of BSA (10, 20, or 40 μg), or the source of the BSA (template or primer pools). Amplicon mt_3 again dropped out in some of the samples with BSA; it is unknown if this is due to the primer binding issue or partial inhibition. These results show that non-specific adaptor ligation of nhDNA occurs due to the presence of both inhibitors and microbial DNA and that BSA can overcome the inhibition, allowing the human mtDNA to be amplified and sequenced even when the quantity of non-human, microbial DNA relative to human DNA is overwhelming.

### 3.3. Bone Casework Samples

**Figure 7:**
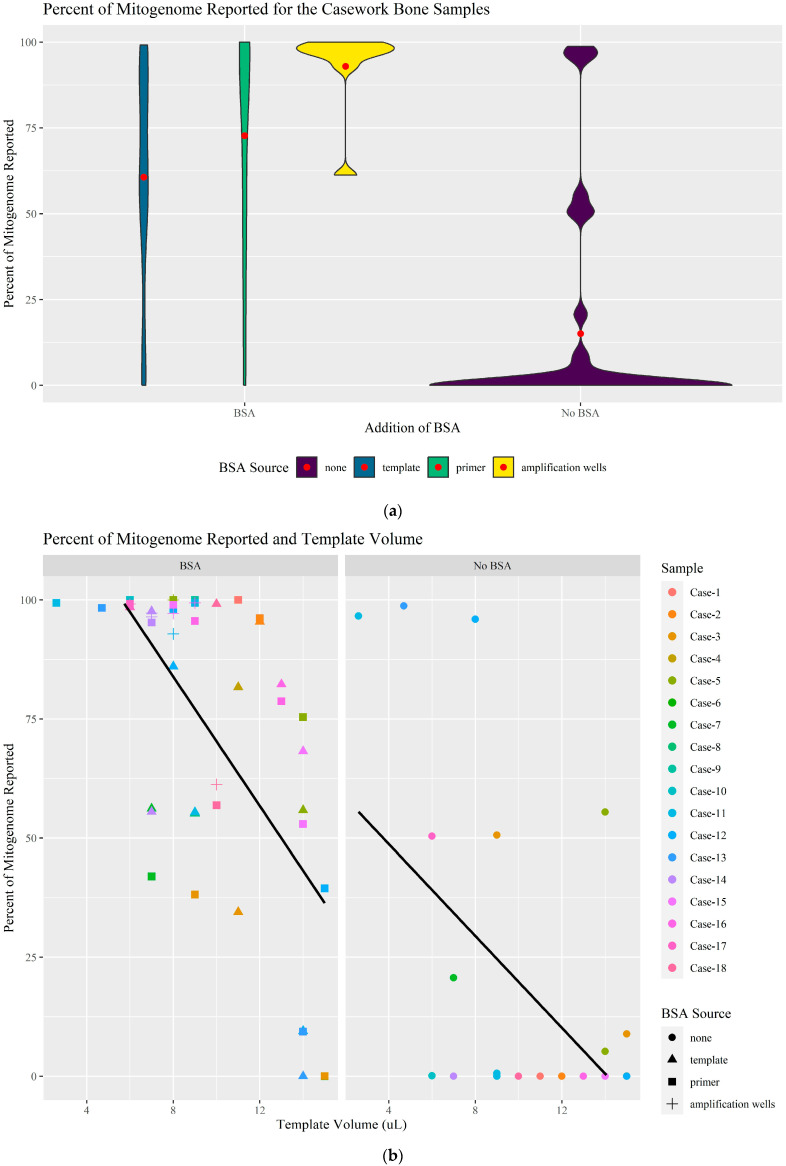

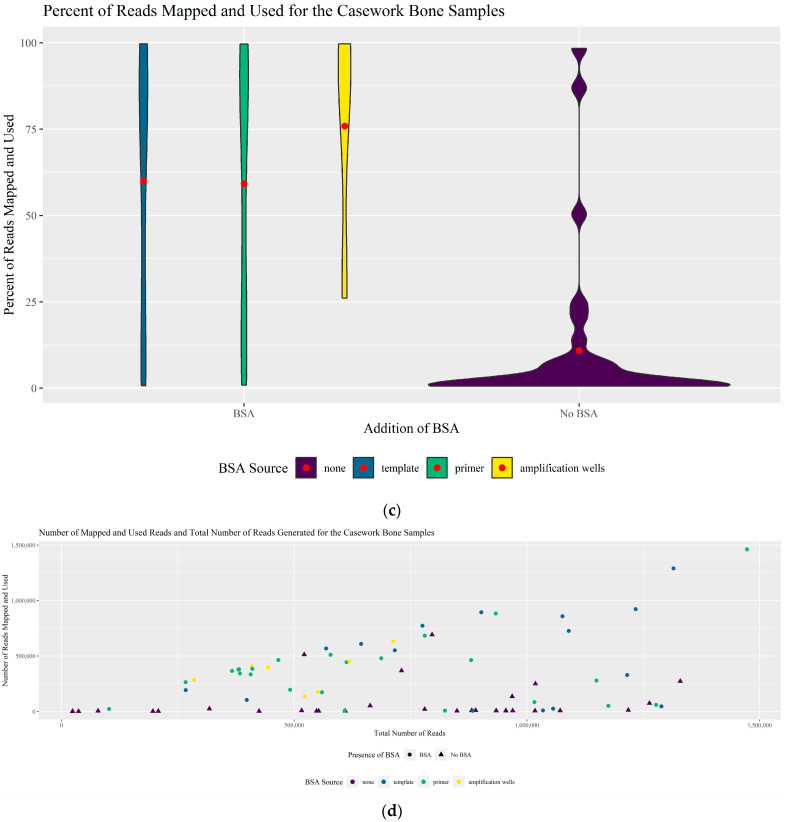
(**a**) Violin plot for the comparison of the percent of the mitogenome reported with and without BSA. The red dot represents the mean. (**b**) Scatter plot of the percent of the mitogenome reported and the template volume used. The solid black line is the trend line. (**c**) Violin plot for the comparison of the percent of mapped and used reads with and without BSA. The red dot represents the mean. (**d**) Scatter plot of the number of reads mapped and used compared to the total number of reads generated.

The effect of BSA on 18 MPDP bone samples exhibiting “whale-pattern” read-length histograms was assessed by evaluating the percent of the whole mitogenome recovered/reported, the percentage of usable mapped reads, and the haplotype concordance. BSA was tested at 20 μg per reaction and added to either the template, the primer pools, or the amplification wells of the IonCode plate. Sixteen of the eighteen MPDP bone samples tested were helped in some capacity by the addition of BSA (Figure 7; Table S1, Supplementary Materials). Twelve of the bones were tested multiple times with varying template volumes and/or BSA sources, resulting in a total of 80 extract aliquots sequenced (32 without BSA and 48 with BSA) (Table 4; Table S1, Supplementary Materials). On average, 71.83% of the whole mitogenome reached a read coverage above the CT with the addition of BSA. Figure 7a shows the distribution of the percentage of the mitogenome reported; while a wide range in coverage occurred for samples with and without BSA, based on the data presented via the violin plots and the read-length histograms in Table 5, the addition of BSA was beneficial in overcoming at least some of the inhibition. Of the 18 MPDP bone samples, 15 had a comparable profile obtained by MPDP caseworkers when they either used a lower template volume, performed additional extract purification, and/or tested a different sample from the same individual. It is common practice in forensic casework, when inhibition is suspected, to lower the amount of template DNA, concomitantly decreasing the amount of inhibitor. Figure 7b illustrates that, by decreasing the template volume of some of the samples, more of the mitogenome was reported regardless of whether BSA was present or not. In this study, a combination of lowering the template volume and adding BSA sometimes resulted in a more complete, interpretable profile than simply adding BSA alone. This demonstrates that BSA is limited in its ability to overcome inhibition and is likely dependent on the inhibitor concentration.

As discussed in Section 3.2, the low number of usable mapped reads compared to the number of total reads generated, along with the “whale-pattern” read-length histogram, are indicators of the presence of nhDNA and an inhibitor interfering with the human mtDNA. When sufficient samples were available, the total DNA quantity was determined using a fluorometer. On average, the amount of nhDNA was 75,000× greater than the amount of human DNA (see Table S1, Supplementary Materials for all nhDNA: human DNA ratios and quantities). Without BSA, the bone samples resulted in an average of 10.92% of the reads generated being mapped to the mitogenome while with BSA, that average increases to 62.29%. Based on these values and the data presented in Figure 7c, BSA was not as successful at increasing the number of usable mapped reads as it was in the previous studies (Section 3.1 and Section 3.2); however, it still resulted in higher quality data comparatively. The data in Figure 7d further supports the claim that non-specific adapter ligation of nhDNA is occurring; for most of the samples without BSA, the number of total reads generated reached upwards of 500,000 reads while the number of reads that were aligned to the mitogenome were usually less than 100,000. For example, 1,017,105 total reads were generated for sample Case-12, but only 6499 reads were used (0.64% read usage). Furthermore, a “whale-pattern” histogram was observed for all 18 bone samples—except when lower template volumes were used for samples Case-11 and Case-13—without the addition of BSA (Table 5; Table S1, Supplementary Materials). With BSA, while not all the histograms have the expected “successful” pattern illustrated in Figure 1b, an improved histogram pattern and a higher percentage of the mitogenome reported were achieved for 16 of the 18 bones.

When possible, the mtDNA haplotypes generated from each of the 80 sequenced samples were compared to the PID sequence previously obtained by MPDP. Three of the bones (Case-1, Case-2, and Case-3) were previously observed by MPDP to contain mixtures and/or poor-quality degraded DNA sequences with NUMTs and damage, such as deamination. Therefore, sequences generated from these bone samples during this study could not be assessed for concordance. Bones Case-4 and Case-5 were also degraded with NUMTs and mixed sites, but partial profiles were obtained by MPDP suitable for comparison. The analyzed haplotypes of all samples amplified and sequenced with BSA were concordant, with differences in amplicon coverage. The only exception was Case-6, which was still fully inhibited. The profiles for Case-5 with BSA also contained poor-quality DNA, and only the variants with “confirmed” status in the analysis software (HIDGenotyper 2.2/Mito IGV 1.09b) were considered. Sample Case-7 contained a low-level mixture; however, the major profile was concordant with the expected profile. Overall, the addition of BSA resulted in concordant profiles.

When assessing each BSA source individually, adding BSA to the amplification wells directly performed the best on average (Figure 7a,c). When considering the percentage of the mitogenome reported (Figure 7a), adding BSA to the primer pools was the second best, though the percentage of mapped and used reads (Figure 7c) were similar when BSA was added to the template or the primer pools (60.03% vs. 59.21%). The lower success of adding BSA to the template was due to the dropout of the amplicons from primer pool one or two. The cause of the successful amplification with one primer pool and failed amplification with the other is unknown; however, the loss of amplicons from one of two primer pools has been previously observed [11,15]. Cuenca et al. [11] speculated that this was due to a bubble preventing the automated pooling of the PCR products of one of the two multiplex reactions. In this study, the loss of one amplicon pool may be due to incomplete mixing of BSA in the template, and therefore, non-uniform distribution of BSA to the amplification wells (either column two [primer pool one] or column three [primer pool two]) during the transfer of the template from column one. While adding the BSA to the amplification wells directly performed the best, the number of required pipetting steps (16 steps) during the manual plate set-up makes it less desirable. Adding the BSA to the primer pools, with only two pipetting steps, was determined to be the preferable method.

## 4. Conclusions

The PID mtDNA system is sensitive to inhibitors and microbial DNA co-extracted from human remains. Based on the findings of the current study, the addition of BSA to the PID mtDNA system improved the robustness of the system overall. The effectiveness of BSA is inhibitor-concentration dependent, and while an upper limit of the benefit is unknown, BSA was effective for templates containing at least 50 ng/μL humic acid, 50 μM hematin, and 1500 μM Ca^2+^. Additionally, BSA resulted in at least partial mitogenomes for 16 of the 18 inhibited bone casework samples that previously generated no results. The results of this study also showed that non-specific adaptor ligation of non-human DNA occurs when both an inhibitor and microbial DNA are present in an extract and that BSA is capable of alleviating the inhibition, allowing the human mtDNA to be amplified and sequenced even when the majority of the sample is composed of non-human, microbial DNA.

BSA can be added to multiple components of the library preparation step; our recommendation is to add 20 μg of BSA per amplification reaction to the primer pools of the panel to avoid uneven distribution of the BSA and decrease the risk of contamination. BSA was shown to not be detrimental to the effectiveness of the PID mtDNA system alone and could therefore be routinely added as an approach to preemptively minimize PCR inhibition.

## Figures and Tables

**Table 1:** Settings for analysis of sequencing data.

	Number of Samples Multiplexed		
	16	24	32
Analytical Threshold *	1.25%	0.83%	0.63%
Minimum Total Read Coverage per Base Position	250	167	125
Minimum Variant Coverage to Call	25	17	12.5
Coverage Threshold	250	167	125
Minimum Coverage Percent Compared to the Median of the Amplicon	5%	5%	5%
Confirmed Call Threshold	90%	90%	90%
Point Heteroplasmy Call Threshold	10%	10%	10%
Insertion Call Threshold	20%	20%	20%
Deletion Call Threshold	30%	30%	30%

* The analytical threshold is a setting not built into the Torrent Suite Software 5.10.0 nor the IGV 1.09b; it was established by the CA DOJ MPDP and is run-specific. For each run, a sample’s percentage of the total usable reads in the run is determined, and if it does not meet the analytical threshold, it is not analyzed further [11].

**Table 2:** Precision ID read-length histograms for QC. Included are QC samples without inhibitor and with the highest amount of each inhibitor tested. The amount of BSA is listed below the histogram along with the source of the BSA in parentheses.

Sample	No BSA	BSA
QC	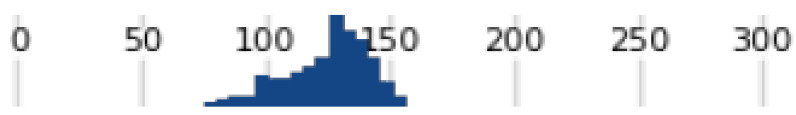	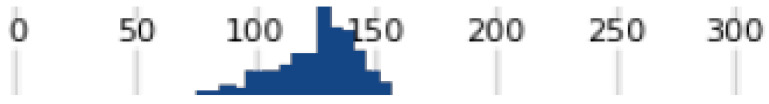 10 μg (template)
QC + 50 ng/μL Humic Acid	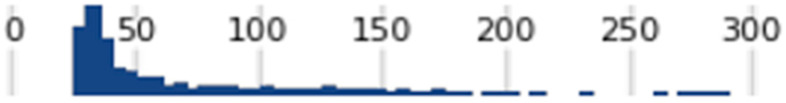	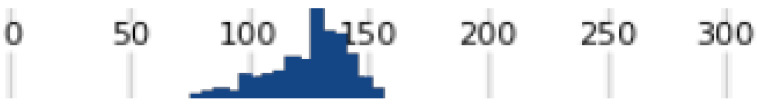 20 μg (primer)
QC + 50 μM Hematin	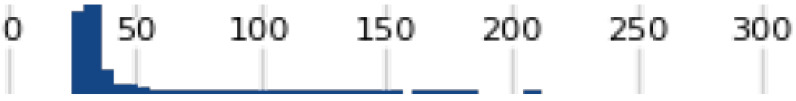	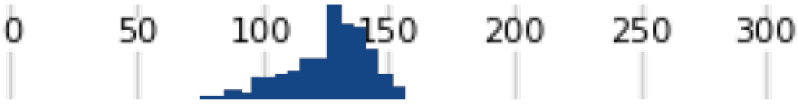 20 μg (primer)
QC + 1500 μM Calcium	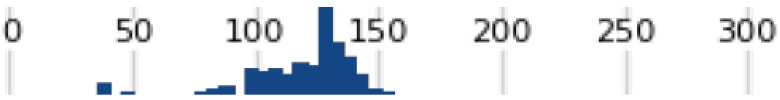	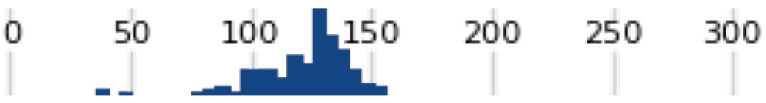 20 μg (primer)

**Table 3:** Precision ID read-length histograms for QC with nhDNA. The amount of BSA is listed below the histogram along with the source of the BSA in parentheses.

Sample	No BSA	BSA
QC + 100 ng nhDNA	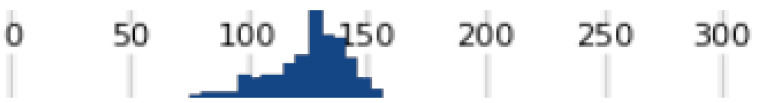	N/A
QC + 100 ng nhDNA + 10 μM Hematin	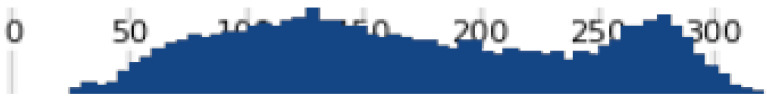	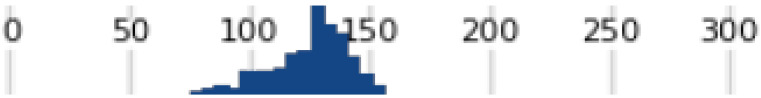 10 μg (template)
QC + 1000 ng nhDNA + 10 μM Hematin	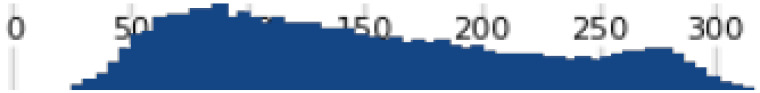	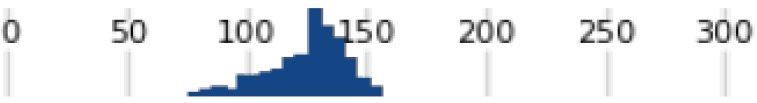 20 μg (primer)
QC + 1000 ng nhDNA + 50 μM Hematin	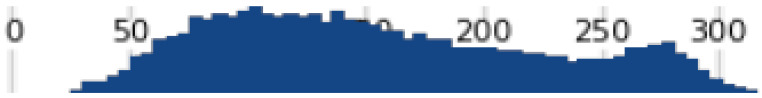	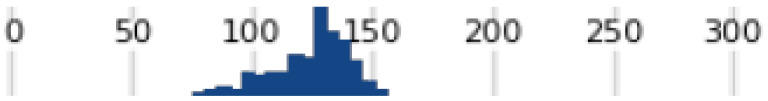 20 μg (primer)
QC + 1000 ng nhDNA + 50 ng/μL Humic Acid	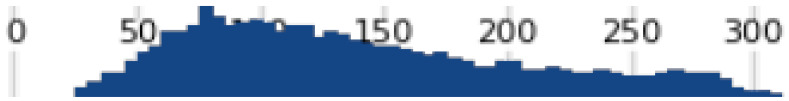	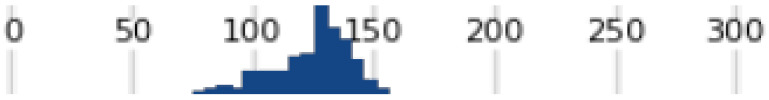 20 μg (primer)

**Table 4:** Casework bone samples. This table includes the sample name, the extract number, template human DNA quantity amplified, in picograms (pg), non-human (nh) DNA quantity amplified, in nanograms (ng), template volume used, and presence/absence of BSA. “Both” indicates that aliquots of the same volume from the same quantified extract were run both with BSA (“Yes”) and without BSA (“No”). Some bones were extracted multiple times when not enough extract volume was available for all experiments. Samples with N/A were not tested for nhDNA.

Sample Name	Extract Number	Template Human DNA Quantity Amplified (pg)	nhDNA Quantity Amplified (ng)	Template Volume Used (μL)	BSA
Case-1	1	55.1 *	35.1	11	Both
Case-2	1	3.6 *	156.0	12	Both
	2	8.0 *	336.0	12	Both
Case-3	1	22.5 *	N/A	15	No
	1	16.5 *	N/A	11	Yes
	2	9.8	N/A	15	Both
	2	5.9	N/A	9	Both
Case-4	1	11.2	N/A	15	No
	1	8.2	N/A	11	Yes
Case-5	1	24.7	N/A	14	Both
	1	14.1	N/A	8	Yes
	2	12.0	140.0	14	Both
Case-6	1	2.6	3808.0	14	Both
Case-7	1	13.7	567.0	7	Both
	2	77.3 *	5054.9	15	Both
	2	36.1 *	2359.0	7	Both
Case-8	1	335.7	48.3	9	Both
Case-9	1	21.1	486.0	9	Both
Case-10	1	173.6	389.8	6	Both
Case-11	1	377.6	674.6	9	Both
	2	100.0 ⁺	139.9	2.6	Both
	2	345.9 ⁺	485.7	9	Both
Case-12	1	121.0	N/A	14	Both
	1	69.2	1127.9	8	Yes
	2	69.5	2114.9	15	Both
	2	37.1	1128.0	8	Both
Case-13	1	503.5	3429.5	14	Both
	2	100.0 ^+^	944.9	4.7	Both
	2	455.8	2813.5	14	Both
Case-14	1	371.0	90.6	7	Both
Case-15	1	337.2	N/A	14	Both
	1	192.7	N/A	8	Yes
	2	269.3	58.5	14	Both
Case-16	1	444.0	N/A	13	Both
	1	307.4	N/A	9	Yes
	2	352.4	1533.6	13	Both
Case-17	1	871.4	1757.1	6	Both
Case-18	1	551.4	3939.4	10	Both

* Small target only; ⁺ Large target only.

**Table 5:** Precision ID read-length histograms for the casework bone sample extract aliquots tested with and without BSA, using the same volume. The read-length histograms for all of the sequenced aliquots are in Table S1, Supplementary Materials. Below each histogram is the percentage of the mitogenome reported.

**Sample**	**Template Volume and Extract Number**	**No BSA**	**BSA Source**	**BSA**
Case-1	11 μL Extract 1	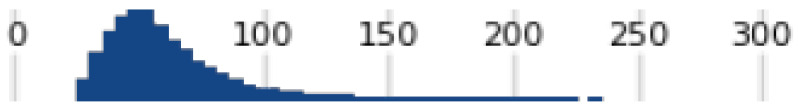 0%	primer	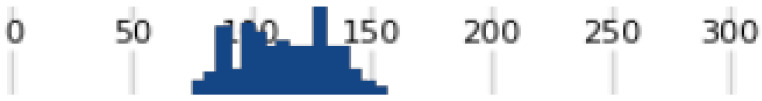 100%
Case-2	12 μL Extract 2	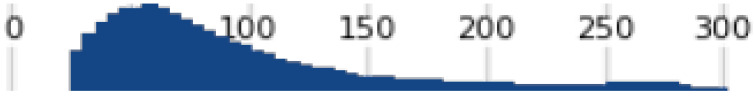 0%	primer	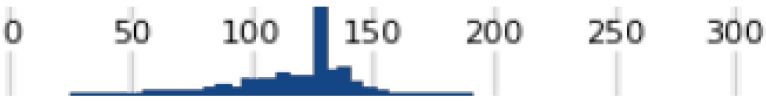 96.15%
Case-3	9 μL Extract 2	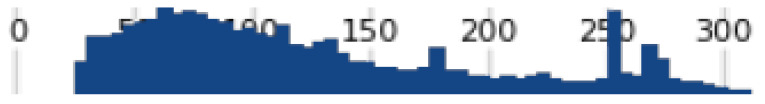 50.62%	primer	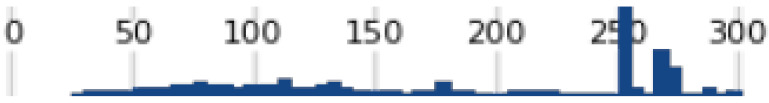 38.16%
	15 μL Extract 2	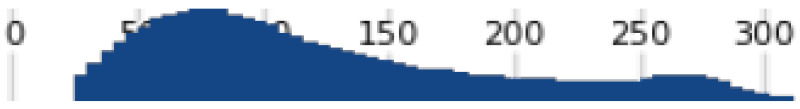 0.02%	primer	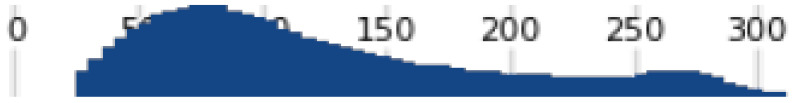 0.02%
Case-4	11 μL Extract 1	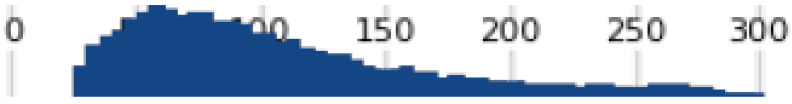 0%	template	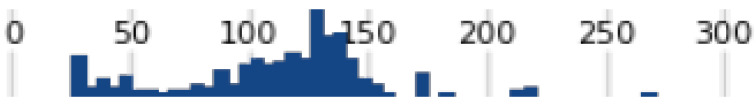 81.69%
Case-5	14 μL Extract 2	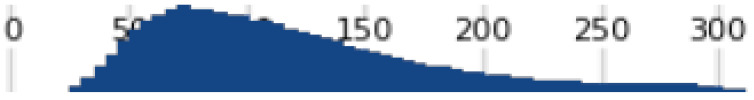 5.20%	primer	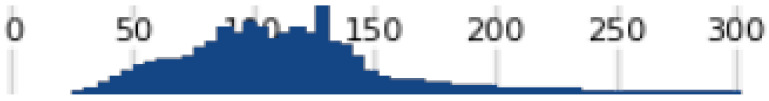 75.40%
Case-6	14 μL Extract 1	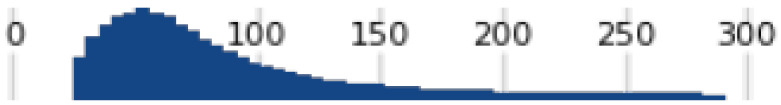 0%	template	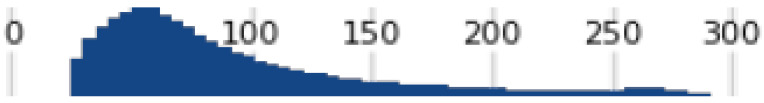 0%
Case-7	7 μL Extract 2	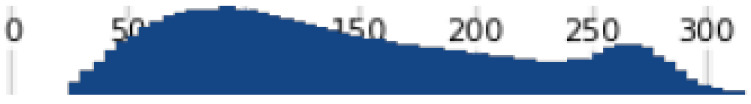 0%	primer	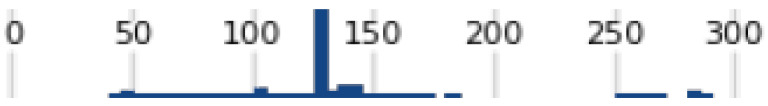 41.96%
	15 μL Extract 2	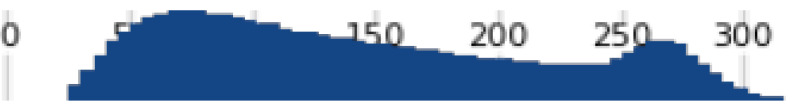 0%	primer	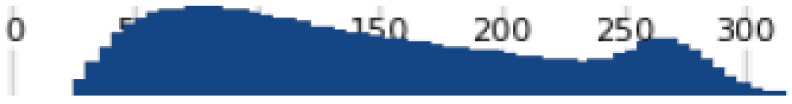 0%
Case-8	9 μL Extract 1	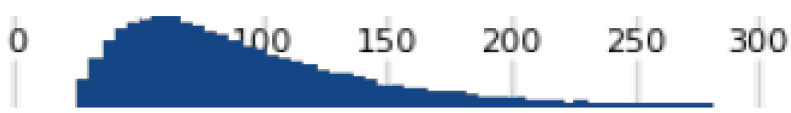 0%	template	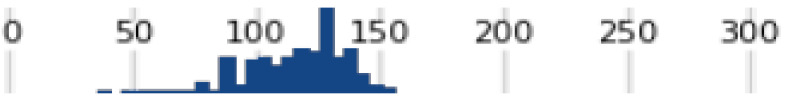 55.13%
Case-9	9 μL Extract 1	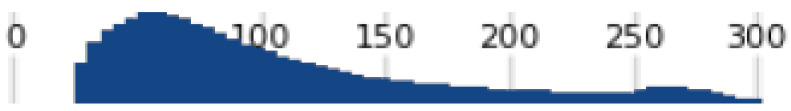 0.04%	primer	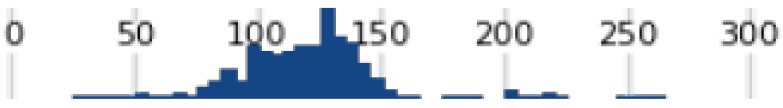 100%
Case-10	6 μL Extract 1	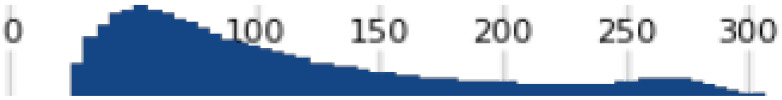 0.08%	primer	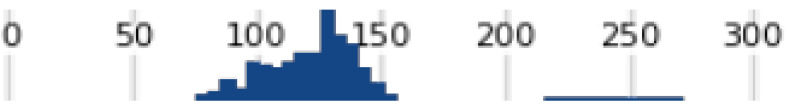 100%
Case-11	9 μL Extract 2	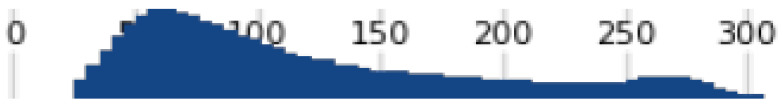 0%	primer	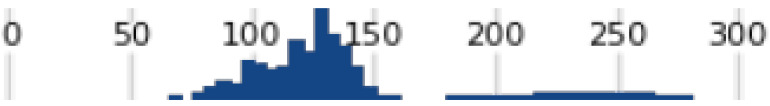 99.38%
	2.6 μL Extract 2	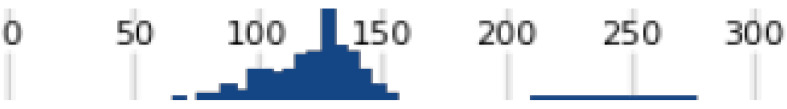 96.61%	primer	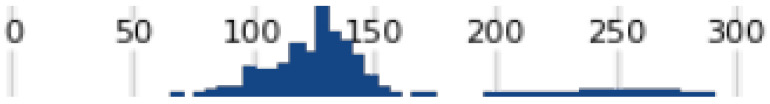 99.38%
Case-12	15 μL Extract 2	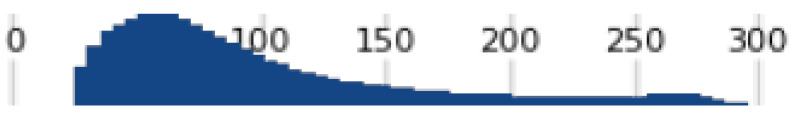 0%	primer	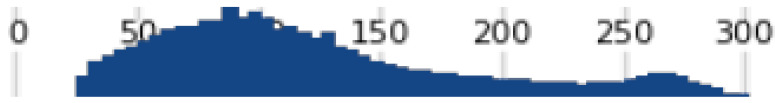 39.44%
	8 μL Extract 2	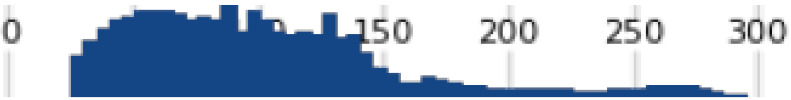 95.94%	primer	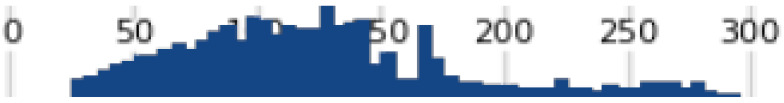 98.10%
Case-13	4.7 μL Extract 2	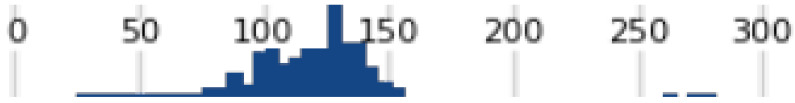 98.74%	primer	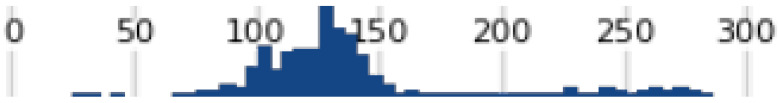 98.33%
	14 μL Extract 2	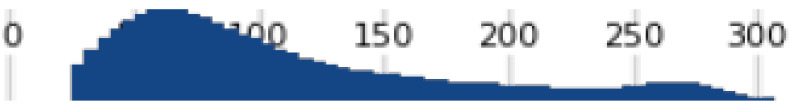 0%	primer	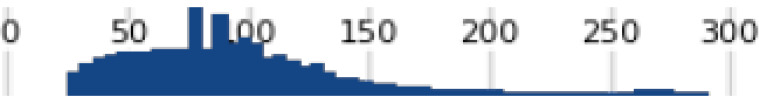 9.37%
Case-14	7 μL Extract 1	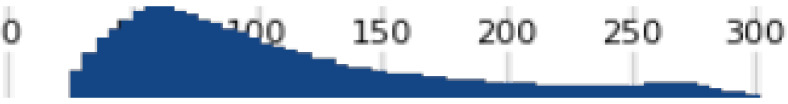 0%	primer	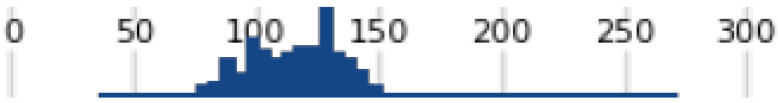 95.27%
Case-15	14 μL Extract 2	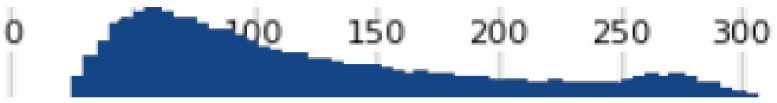 0%	primer	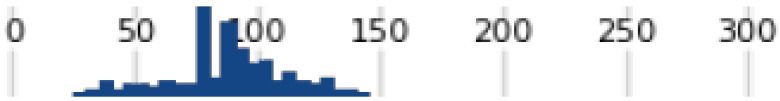 52.92%
Case-16	13 μL Extract 2	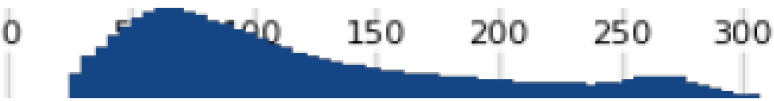 0%	primer	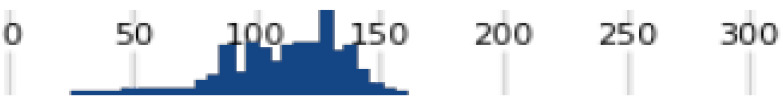 78.74%
Case-17	6 μL Extract 1	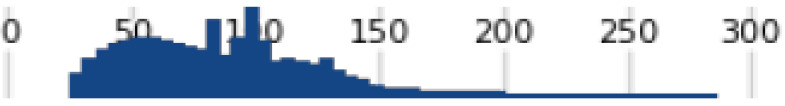 50.37%	primer	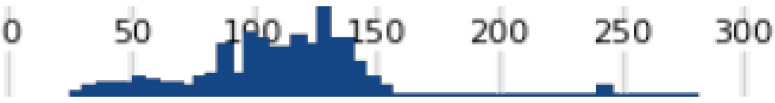 99.20%
Case-18	10 μL Extract 1	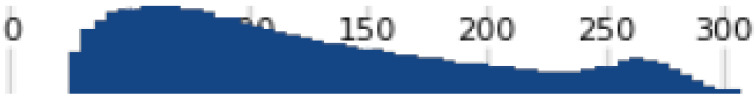 0%	primer	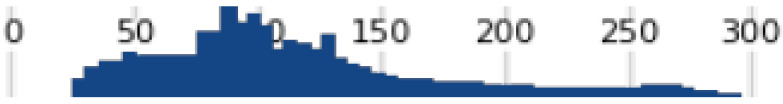 56.87%

## Data Availability

Some of the data presented in this study are available upon request from the corresponding author. Some of the data in this study are restricted due to privacy concerns and are not available.

## Supplementary Materials

The following supporting information can be downloaded at: https://www.mdpi.com/article/10.3390/genes16020119/s1, Figure S1: Percent of mitogenome reported and percent of reads mapped and used for PS1; Figure S2: Normalized amplicon coverage for PS1; Figure S3: Percent of mitogenome reported and percent of reads mapped and used for Bone1; Figure S4: Normalized amplicon coverage for Bone1; Figure S5: Number of mapped and used reads and total number of reads generated for humic acid inhibited samples; Figure S6: Number of mapped and used reads and total number of reads generated for hematin-inhibited Samples; Figure S7: Number of mapped and used reads and total number of reads generated for calcium-inhibited samples; Figure S8: mtDNA linear coverage plots of QC; Table S1: Sample information and sequencing results for the casework bone samples; Table S2: Sequencing results for QC without the addition of inhibitors or microbial DNA; Table S3: Sequencing results for the PS1 sensitivity samples; Table S4: Sequencing results for the Bone1 sensitivity samples; Table S5: Sequencing results of QC for the inhibition study; Table S6: Sequencing results of QC for the microbial DNA study.
