# Supplementary material for: Enhancement of the Precision ID Mitochondrial DNA Whole Genome System for Challenging Unidentified Human Remains
Source: Genes (Basel). 2025 Jan 22;16(2):119. doi: 10.3390/genes16020119 (PMC11855493; doi:10.3390/genes16020119)
Supplement: Supplementary file 1 [file genes-16-00119-s001.zip › Supplementary Figures.pdf]

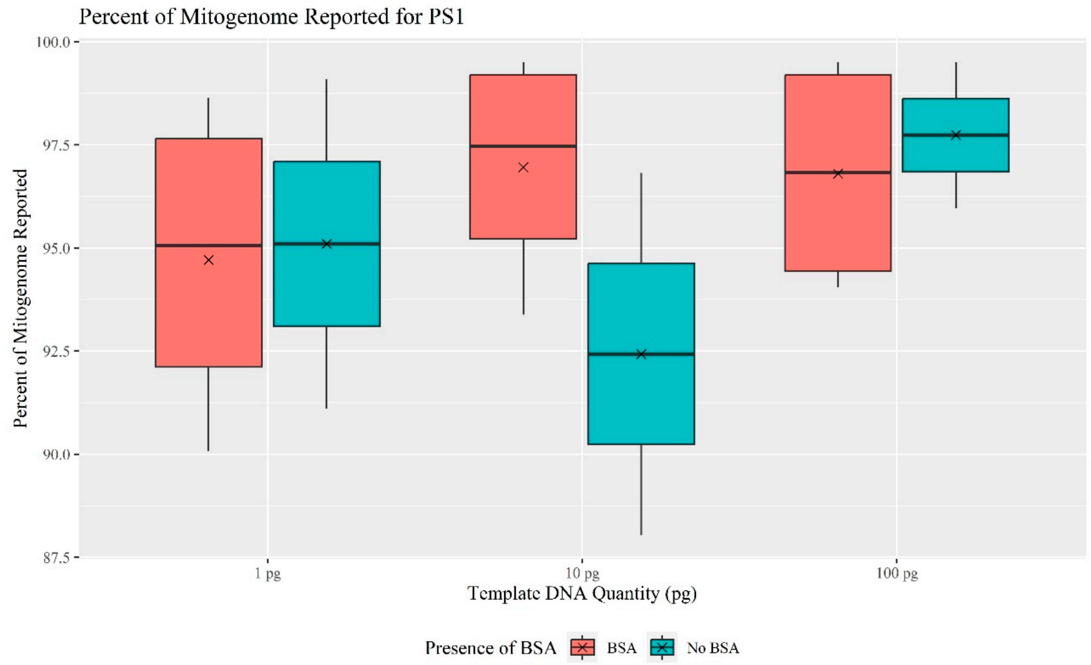

(a)

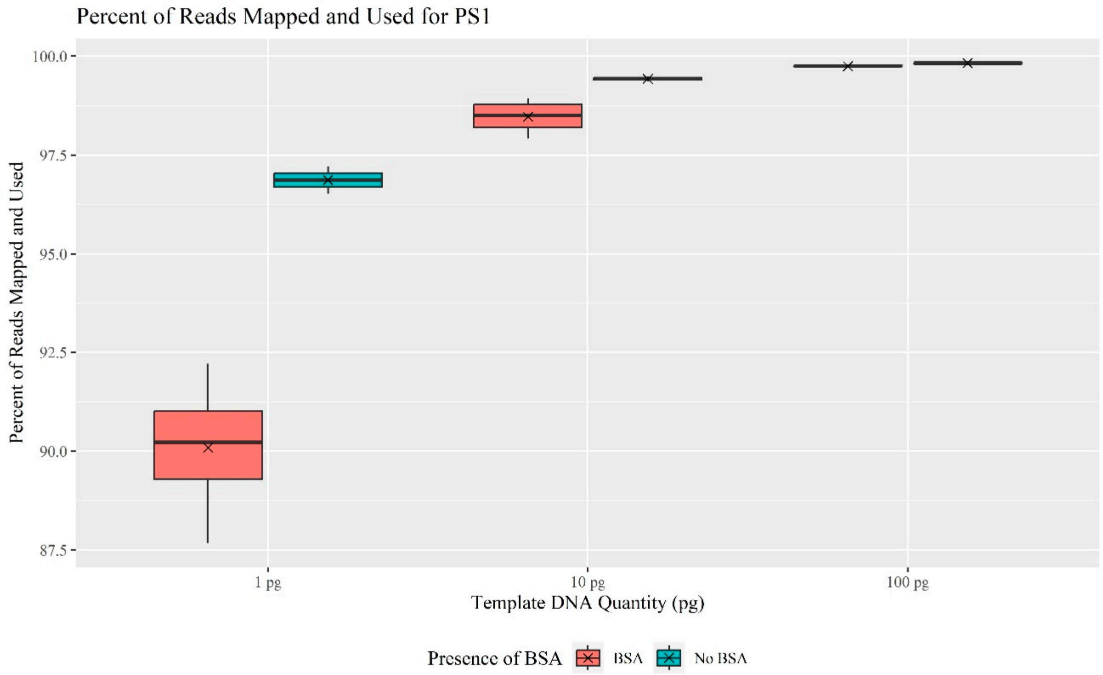

(b)

Figure S1. (**a**) Boxplots for the comparison of the percent of the mitogenome reported with and without BSA for each input amount of PS1. The 'x' represents the mean and the line is the median. (**b**) Boxplots for the comparison of the percent of the mapped and used reads with and without BSA for each input amount of PS1. The 'x' represents the mean and the line is the median.

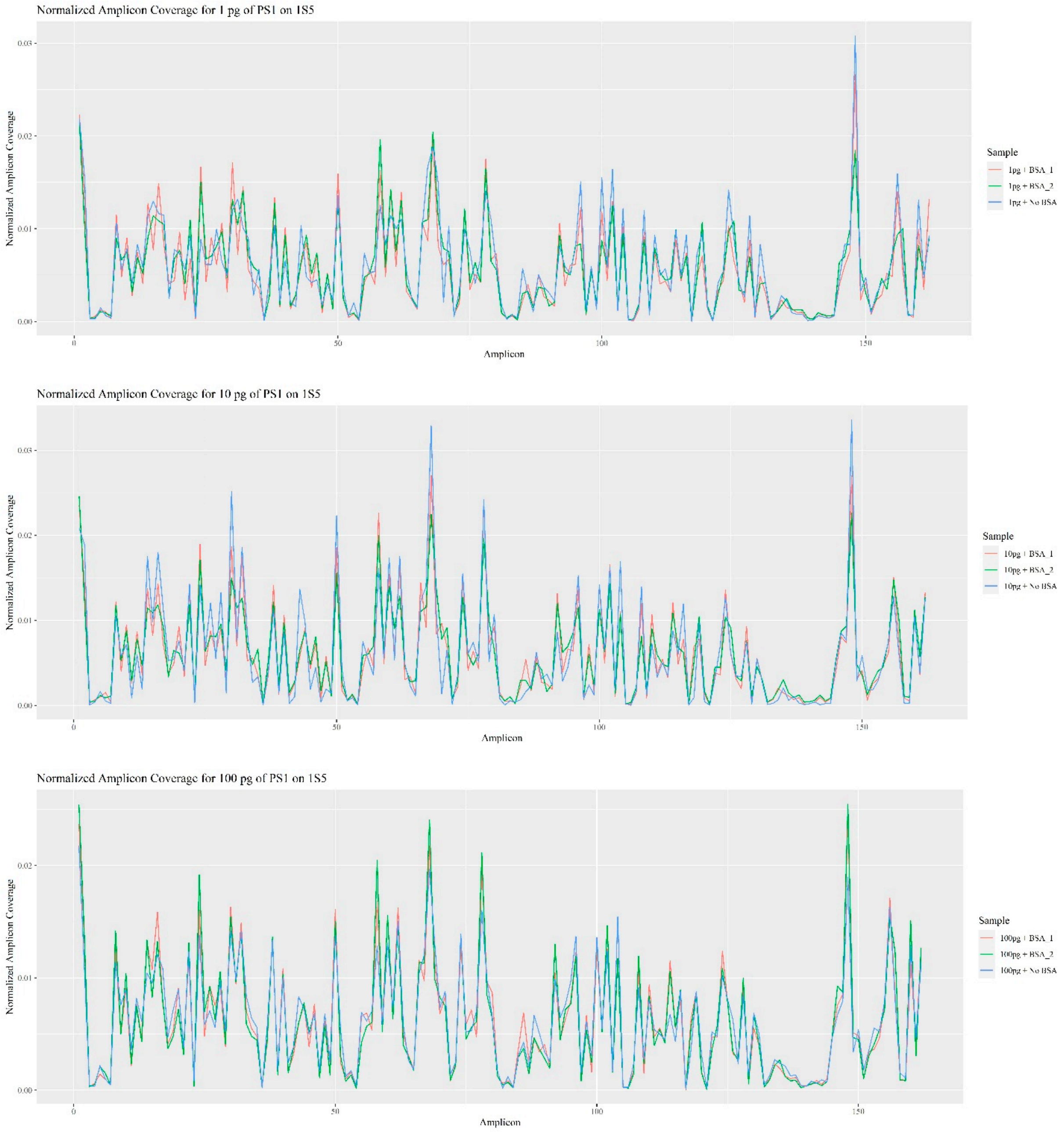
Normalized Amplicon Coverage for 1 pg of PS1 on 1S5
Normalized Amplicon Coverage
0.00
0.01
0.02
0.03
Amplicon
0
50
100
150
Sample
1pg + BSA_1
1pg + BSA_2
1pg + No BSA
Normalized Amplicon Coverage for 10 pg of PS1 on 1S5
Normalized Amplicon Coverage
0.00
0.01
0.02
0.03
Amplicon
0
50
100
150
Sample
10pg + BSA_1
10pg + BSA_2
10pg + No BSA
Normalized Amplicon Coverage for 100 pg of PS1 on 1S5
Normalized Amplicon Coverage
0.00
0.01
0.02
Amplicon
0
50
100
150
Sample
100pg + BSA_1
100pg + BSA_2
100pg + No BSA

(a)

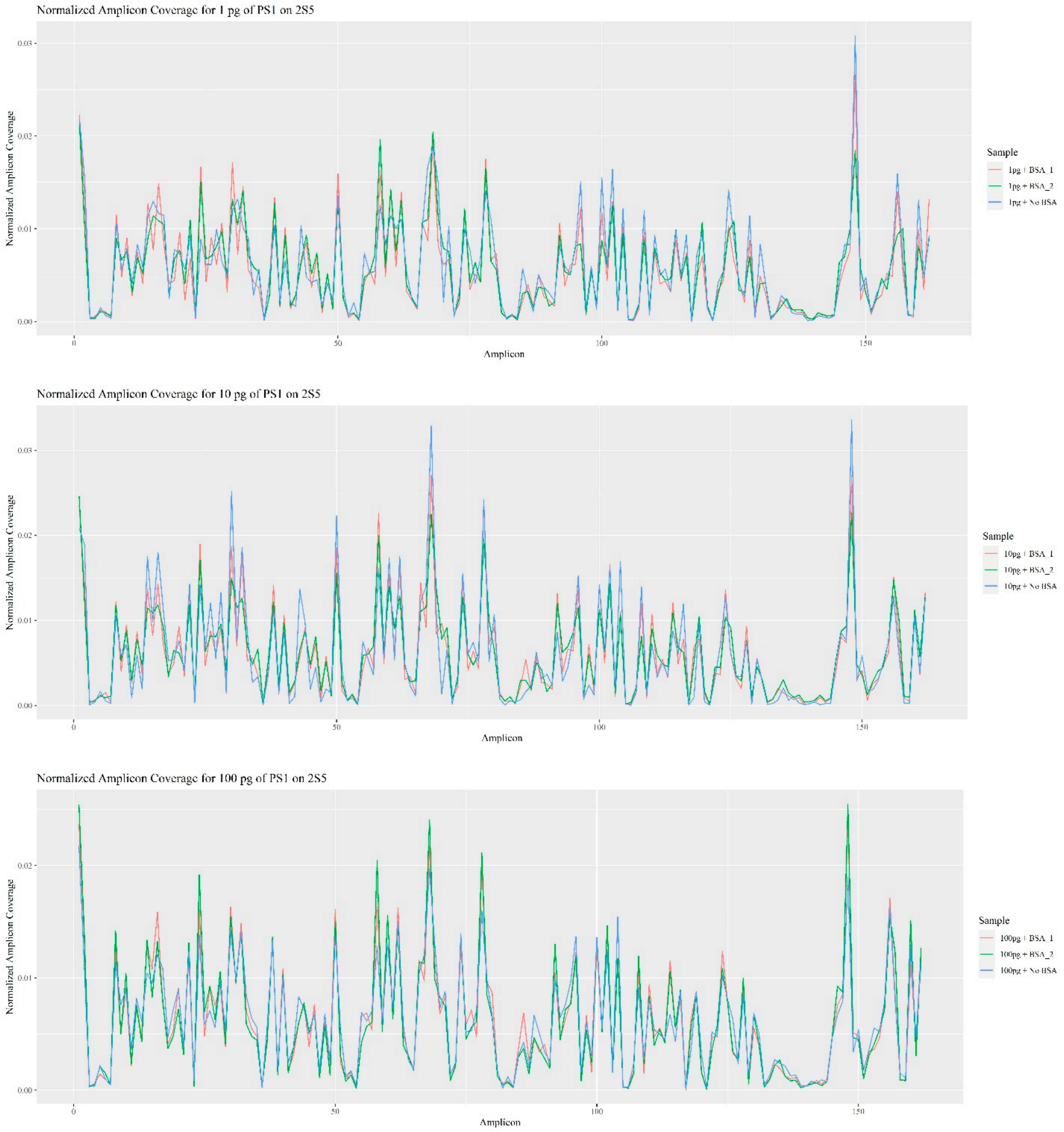

(**b**)

Figure S2. Linear coverage plots for each amplicon at the varying template input amounts for the PS1 sample ran on instrument sets (**a**) 1S5 and (**b**) 2S5.

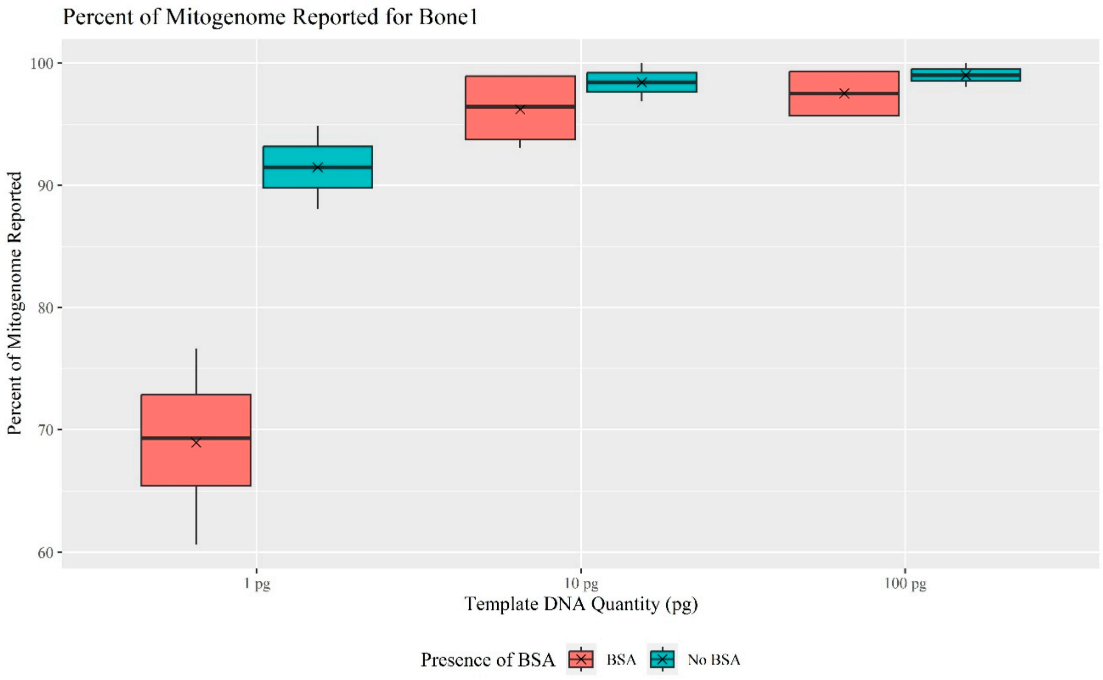

(a)

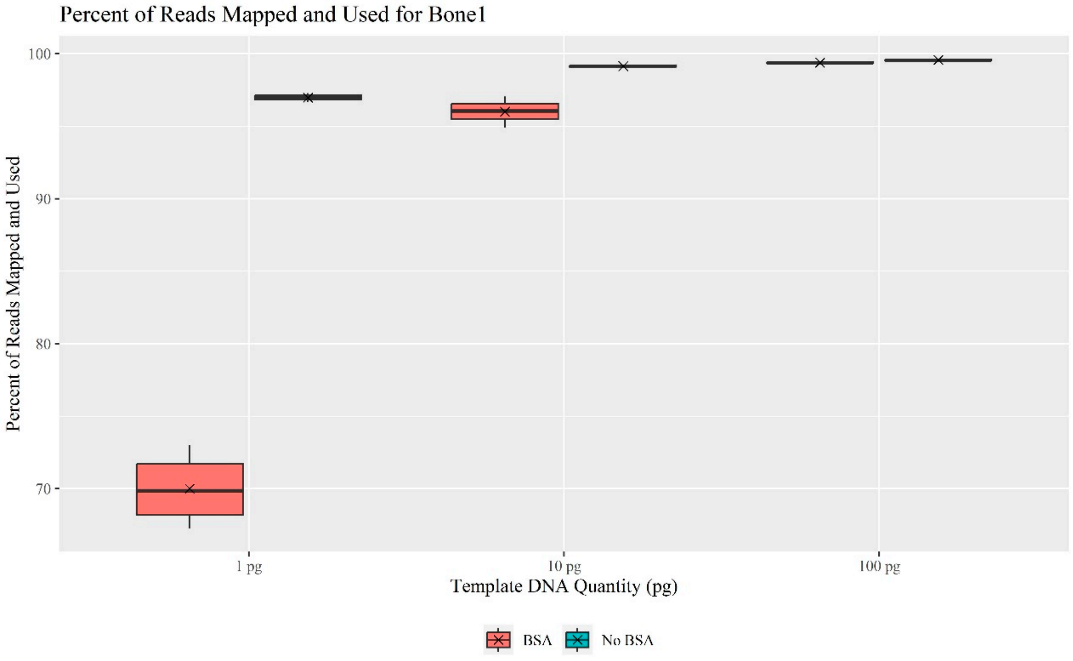

(b)

Figure S3. (**a**) Boxplots for the comparison of the percent of the mitogenome reported with and without BSA for each input amount of Bone1. The 'x' represents the mean and the line is the median. (**b**) Boxplots for the comparison of the percent of the mapped and used reads with and without BSA for each input amount of Bone1. The 'x' represents the mean and the line is the median.

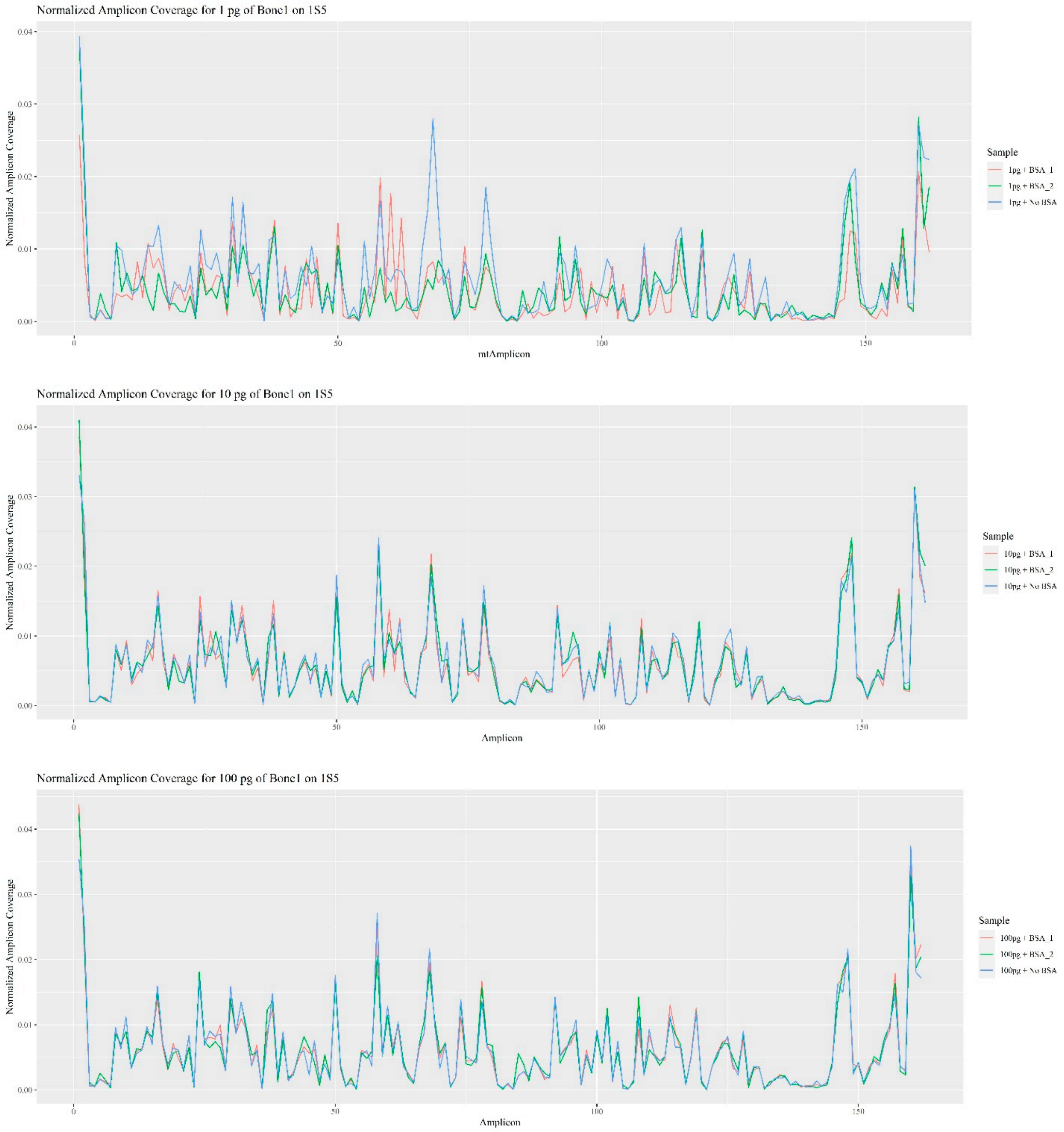
Normalized Amplicon Coverage for 1 pg of Bone1 on 1S5
Normalized Amplicon Coverage
mtAmplicon
Sample
1pg + BSA_1
1pg + BSA_2
1pg + No BSA
Normalized Amplicon Coverage for 10 pg of Bone1 on 1S5
Normalized Amplicon Coverage
Amplicon
Sample
10pg + BSA_1
10pg + BSA_2
10pg + No BSA
Normalized Amplicon Coverage for 100 pg of Bone1 on 1S5
Normalized Amplicon Coverage
Amplicon
Sample
100pg + BSA_1
100pg + BSA_2
100pg + No BSA
0.00
0.01
0.02
0.03
0.04
0
50
100
150

(a)

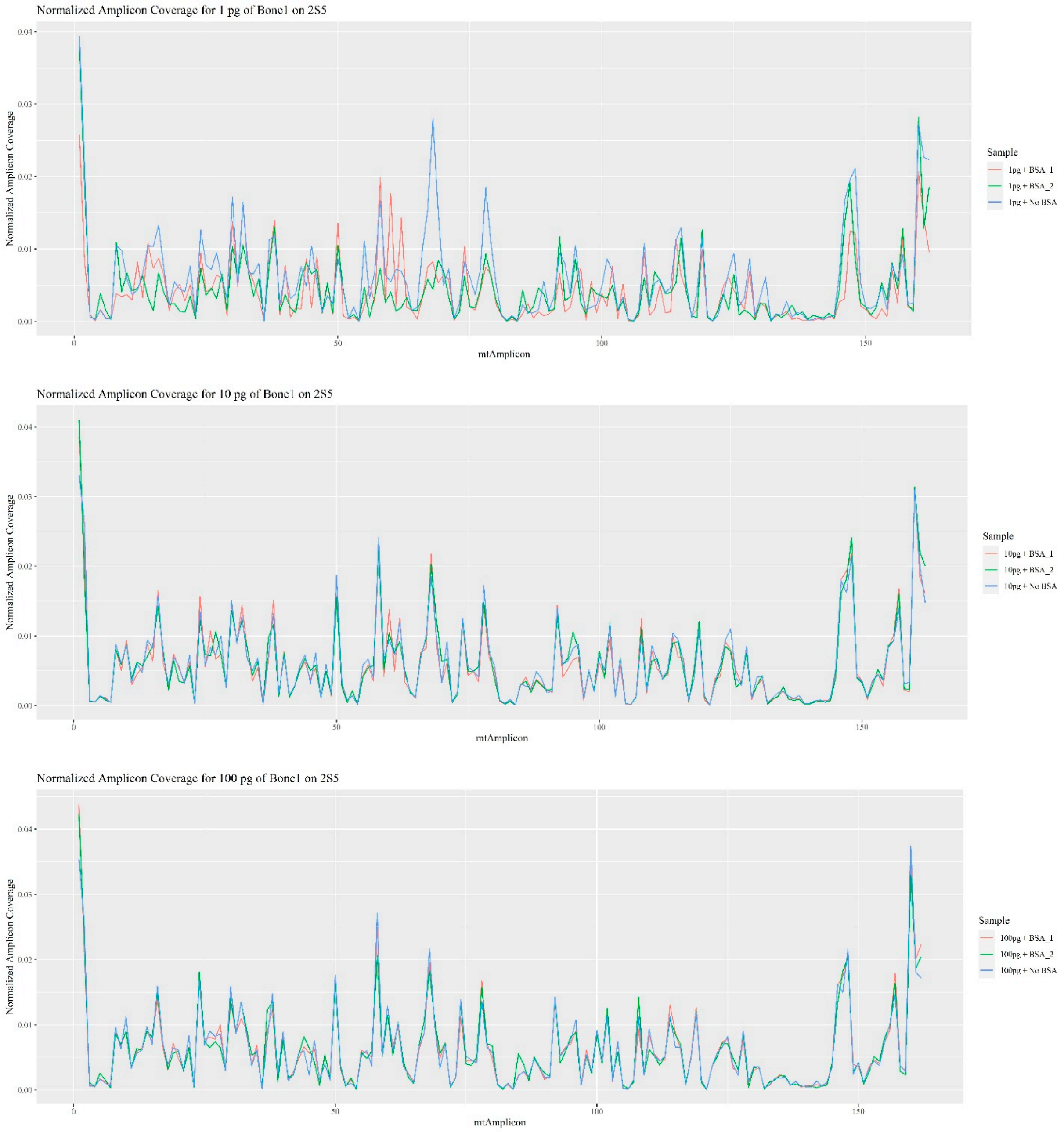

(**b**)

Figure S4. Linear coverage plots for each amplicon at the varying template input amounts for the Bone1 sample ran on instrument sets (**a**) 1S5 and (**b**) 2S5.

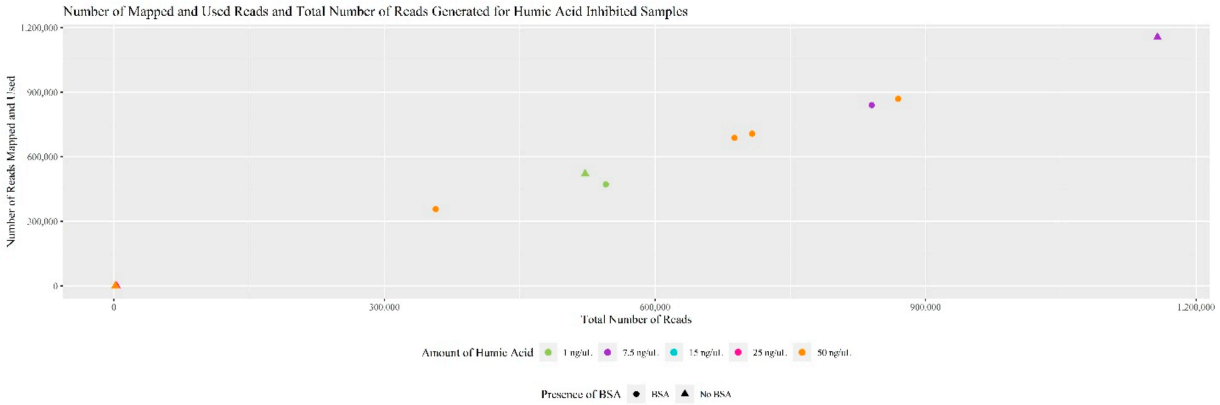

Figure S5. Scatter plot of the number of reads mapped and used compared to the total number of reads generated for each humic acid inhibited sample.

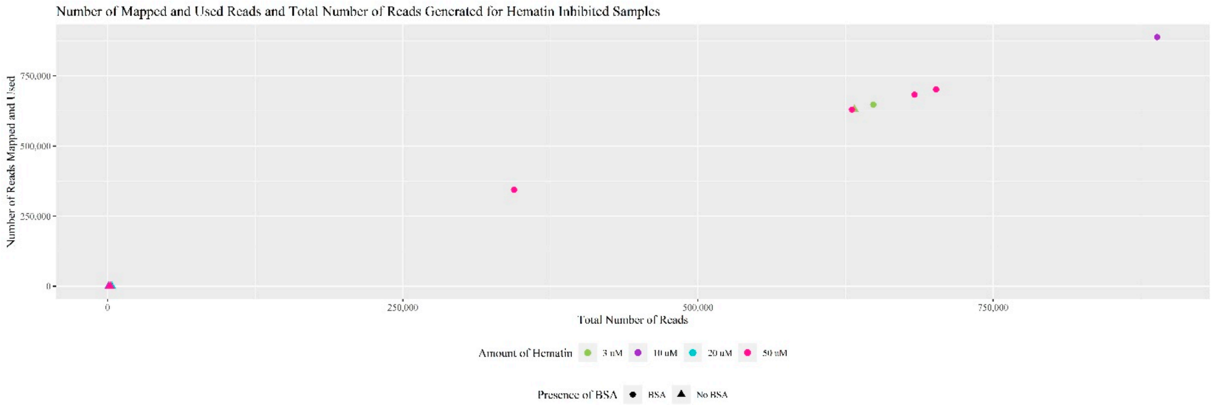

Figure S6. Scatter plot of the number of reads mapped and used compared to the total number of reads generated for each hematin inhibited sample.

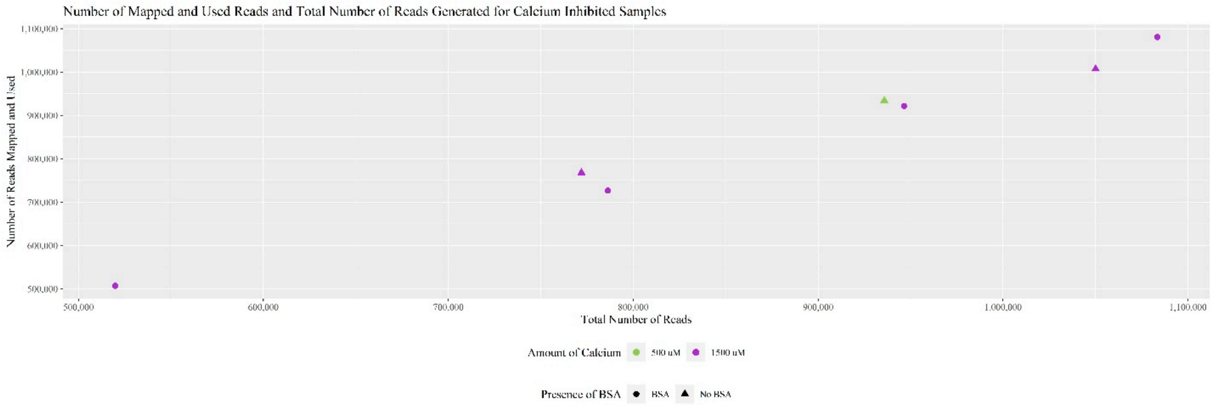

Figure S7. Scatter plot of the number of reads mapped and used compared to the total number of reads generated for each calcium inhibited sample.

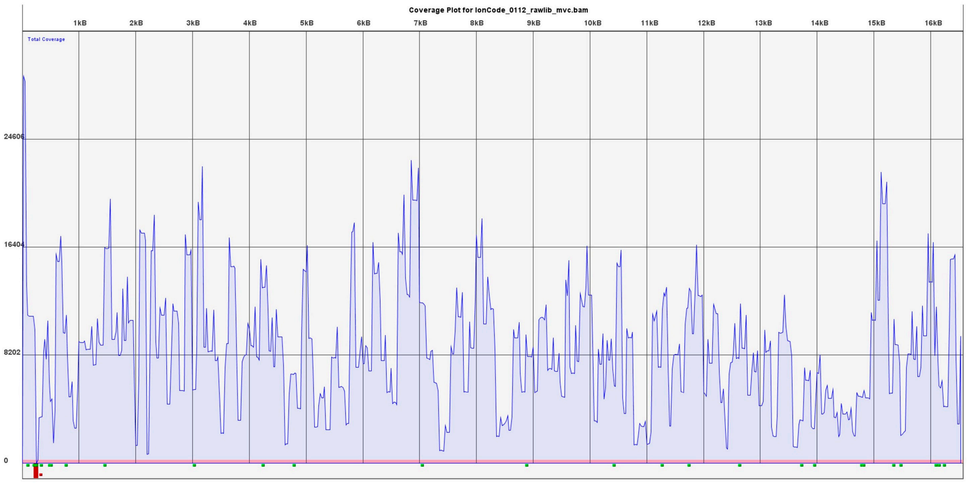

(**a**)

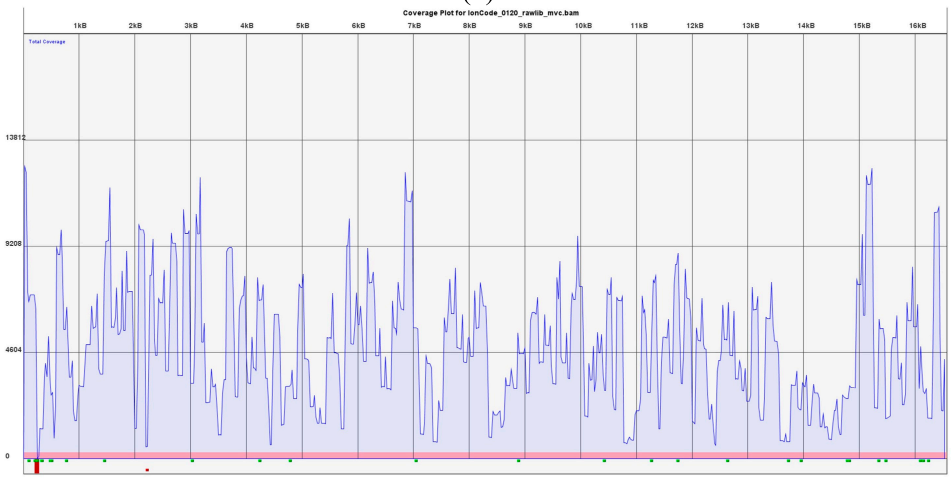

(**b**)

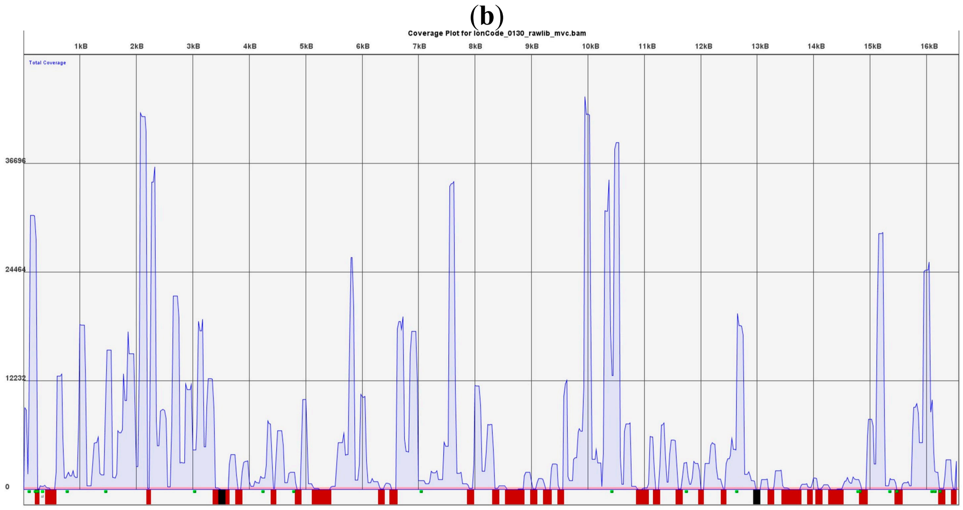

(**c**)

Figure S8. mtDNA linear coverage plots of QC for which partial mitogenomes were detected. Amplifications contained (**a**) 7.5 ng/μL humic acid, (**b**) 3 μM hematin, and (**c**) 1500 μM Ca^{2+}. The x-axis represents SNP positions 1-16,569 of the mitogenome. The y-axis represents the number of usable reads.
